# BiNChE: A web tool and library for chemical enrichment analysis based on the ChEBI ontology

**DOI:** 10.1186/s12859-015-0486-3

**Published:** 2015-02-21

**Authors:** Pablo Moreno, Stephan Beisken, Bhavana Harsha, Venkatesh Muthukrishnan, Ilinca Tudose, Adriano Dekker, Stefanie Dornfeldt, Franziska Taruttis, Ivo Grosse, Janna Hastings, Steffen Neumann, Christoph Steinbeck

**Affiliations:** Cheminformatics and Metabolism, European Molecular Biology Laboratory - European Bioinformatics Institute, Cambridge, UK; Dept. of Stress- and Developmental Biology, Leibniz Institute of Plant Biochemistry, Halle, Germany; Dept. of Statistical Bioinformatics, Institute for Functional Genomics, University of Regensburg, Halle, Germany; Institute of Computer Science, Martin Luther University Halle-Wittenberg, Halle, Germany; German Centre for Integrative Biodiversity Research (iDiv) Halle-Jena-Leipzig, Leipzig, Germany

**Keywords:** Ontology, Enrichment, Small molecules

## Abstract

**Background:**

Ontology-based enrichment analysis aids in the interpretation and understanding of large-scale biological data. Ontologies are hierarchies of biologically relevant groupings. Using ontology annotations, which link ontology classes to biological entities, enrichment analysis methods assess whether there is a significant over or under representation of entities for ontology classes. While many tools exist that run enrichment analysis for protein sets annotated with the Gene Ontology, there are only a few that can be used for small molecules enrichment analysis.

**Results:**

We describe BiNChE, an enrichment analysis tool for small molecules based on the ChEBI Ontology. BiNChE displays an interactive graph that can be exported as a high-resolution image or in network formats. The tool provides plain, weighted and fragment analysis based on either the ChEBI Role Ontology or the ChEBI Structural Ontology.

**Conclusions:**

BiNChE aids in the exploration of large sets of small molecules produced within Metabolomics or other Systems Biology research contexts. The open-source tool provides easy and highly interactive web access to enrichment analysis with the ChEBI ontology tool and is additionally available as a standalone library.

**Electronic supplementary material:**

The online version of this article (doi:10.1186/s12859-015-0486-3) contains supplementary material, which is available to authorized users.

## Background

High-throughput “omics” research produces large sets of data for genes, proteins, and metabolites. Ontology-based enrichment analysis helps to investigate and filter these data sets by assigning meanings to statistical patterns found. Ontologies are hierarchies of biologically relevant groupings such as processes, functions, or activities. Ontology annotations link ontology classes to the biological entities that are known to belong to those groupings or participate in those processes, functions or activities. For example, the Gene Ontology (GO [1]) provides hierarchies of cellular compartments, biological processes, and molecular functions. GO annotations link genes and proteins to those GO classes where the genes are expressed, which processes they participate in, and which functions they bear. Enrichment analysis methods assess whether there is a significant over or under representation of entities for ontology classes, when compared to a background population representing the null hypothesis that the data are not enriched for that class. No classes would be found to be enriched if, for example, the measured biological entities’ annotations were found to be evenly distributed between all available ontology classes, or proportionately according to the known biological distribution for the measured sample (as represented in the proportionality of the background annotation set).

Many enrichment analysis tools have been designed for use with genes or proteins and the GO, including GOMiner, FatiGO, BiNGO and GSEA. For a review, please see Huang *et al.* [2]. These tools take as input lists of gene or protein identifiers and test for enrichment of GO classes against the background of the full set of GO annotations. With the advent of small molecule high throughput data arising from metabolomics – the study of the chemical processes involving metabolites by measuring the presence and quantity of different chemicals in biological samples – similar tools are now needed for small molecules.

ChEBI (Chemical Entities of Biological Interest) is a manually curated database and ontology that organizes small molecule knowledge [3]. ChEBI offers both an ontology of chemical structural classes, such as carboxylic acid and steroid, and a role ontology in which biological and chemical activity classes are organized, such as toxin and solvent. ChEBI also contains fully specifed small molecules at the leaf level of the structural hierarchy – i.e. those that might be measured in metabolomics experiments – such as proline and caffeine. Molecules are linked to structural classes with the *is_a* relation and to functional classes with the *has_role* relation. For example, cefpodoxime (CHEBI:3504) *is_a* carboxylic acid (CHEBI:33575) and *has_role* antibiotic (CHEBI:22582). ChEBI’s chemical ontology straightforwardly allows enrichment analysis techniques to be transferred to the case of small molecules. Here, we present BiNChE, an enrichment analysis tool for the ChEBI ontology. It is available online at http://www.ebi.ac.uk/chebi/tools/binche/ and as open source software libraries (https://github.com/pcm32/BiNChE and https://github.com/pcm32/BiNCheWeb).

BiNChE is not the first chemical enrichment analysis service. MSEA [4] was the first small molecule enrichment analysis service, but it only used collections of small molecules that were considered relevant, rather than chemical ontologies with their richer structure. MBRole [5] was the first small molecule enrichment analysis service to harness ChEBI’s ontology for enrichment. While MBRole now provides a number of different chemical knowledge organization schemes for enrichment analysis, including ChEBI, it displays the results as a plain table, losing the richness of the structure in the underlying ontologies. BiNChE, by contrast, returns graph results and offers a rich visualisation interface. Neither does MBRole include weighted analysis, that is, the ability to influence the enrichment analysis through the inclusion of intensity values, fold-changes or *p*-values as weights for the molecules – an option offered by BiNChE.

Chemical enrichment analysis is also possible with non-dedicated tools. BiNGO [6] is a plugin for Cytoscape that is widely used for protein enrichment analysis and generates interactive graph-based outputs which mimic the structure of the input ontology. BiNGO can be used with ontologies other than the GO, including a chemical ontology, such as ChEBI. However, this requires the manual generation of ontology and annotation files in the custom BiNGO format, which is not straightforward for most users, and the use of the Cytoscape desktop application, rather than being available as a Web utility. The National Center for Biomedical Ontology (NCBO) has also developed an ontology-neutral enrichment analysis tool [7], but that is available exclusively by web service, i.e. there is no associated graphical user interface, and the output it provides is either a tag cloud image (sized for relative frequency) or XML, lacking detail from the source ontology and the statistical significance of the enrichment.

Performing an enrichment analysis with ChEBI has additional subtleties when compared to the GO, further hindering the applicability of generic tools to the chemical case. Firstly, ChEBI includes actual small molecules at the leaf level, while the GO does not include genes or proteins. Thus, ChEBI contains both complex class definitions and content that is analogous to GO annotations. Secondly, while the GO is available in separate files for its three main branches, and in slimmed versions that facilitate enrichment analysis, the ChEBI ontology is only available as a single ontology file that includes the roles and the structural classification. ChEBI also includes parts that are irrelevant for small molecule enrichment analyses, such as sub-atomic particles. All of these issues are solved by BiNChE. In contrast to BiNChE, the use of generic enrichment analysis tools would require manual pre-processing of the ChEBI ontology to remove irrelevant parts of the ontology such as small molecules and sub-atomic particles. Subsequently one would also have to produce a file which annotates each queried ChEBI entity with its ChEBI ontology ancestor terms in order to resemble the use case expected by these tools where proteins are given that have annotated Gene Ontology terms.

## Implementation

BiNChE is implemented in Java version 1.6, starting from the open source code of BiNGO. The application is divided into two projects (with no duplication of code between them): a core engine, which can be used as an standalone desktop application or as a Java library for integration with other projects; and a web application, which displays the enrichment analysis results through an interactive visualization of the ChEBI ontology. The graph based visualization relies on CytoscapeWeb [8] for rendering and provides export functions to generate publication quality images. Results can be also retrieved in tabular and network data formats.

**Figure 1:**
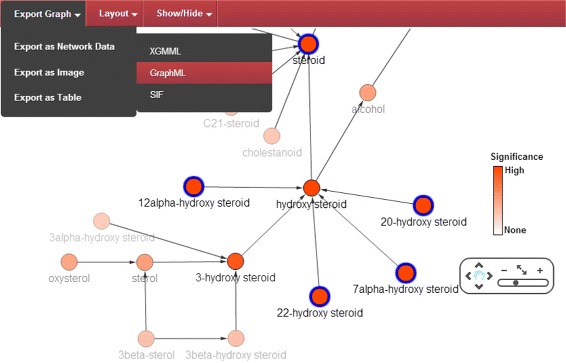
**Screenshot of a result graph from the BiNChE web interface.** Screenshot of a result graph from the BiNChE web interface. Highly enriched nodes are shown in red (increasing transparency as significance decreases). The interface allows selection of nodes (shown in blue) either manually or through right click functions (such as “Select Descendants”). The graph can be exported in different formats, zoomed, and its layout altered manually or through automated algorithms. Nodes and labels can be hidden.

The web-based display of the results (Figure 1) enables the user to modify the resulting enrichment graph through manual re-arrangements and built-in layout algorithms. Nodes representing ChEBI entities display additional information when hovered over. These include the name of the entity, ChEBI identifier, p-value of the binomial test, corrected p-value for multiple hypothesis testing, percentage of the sample that is contained within the ontology term, and the fold of enrichment. The fold of enrichment is the ratio between the enrichment in the sample and enrichment in the background population. Additional color formatting and the ability to selectively hide nodes based on different criteria improves the visualization of interesting parts of the network. A contextual menu with a link to the ChEBI entity web entry, as well as other operations such as the selection of descendants can be triggered by right-clicking the nodes. These features are shown in detail in the wiki (https://github.com/pcm32/BiNCheWeb/wiki/BiNChE#graphical-exploration-of-results) and in Additional file 1.

The tool performs three main types of enrichment analysis: weighted, plain (i.e. unweighted) and fragment. The plain analysis requires a list of ChEBI identifiers to be submitted, and relies on a binomial test to define whether the provided list is enriched for any ChEBI classes. The test is executed – as in any ontology-based enrichment analysis – for each class in the ontology and can be subdivided into a couple of steps that result in a p-value for each term.

A ‘trial’ corresponds to checking whether one of the queried ChEBI identifiers belongs to the ChEBI class being tested. A ‘success’ is registered when the queried ChEBI identifier does belong to the ontology class. The probability of success is given by the total number of possible ChEBI identifiers that belong to that particular ChEBI ontology class, divided by the total number of classes of the ChEBI ontology. It is noteworthy that this implies that the same number of successes within two different ChEBI ontology classes does not guarantee the same level of enrichment, as the level of enrichment also depends on the population-based probability.

For the weighted analysis, the tool accepts as input a list of ChEBI identifiers accompanied by real numbers from 0 to 1 – the *weights* – which can represent intensities or any measurement that reflects differential relevance of the small molecules in an experiment. This type of enrichment uses an implementation of the SaddleSum algorithm [9], which assesses the significance of a *weight* accumulation within an ontology class, drawing a background weight distribution from the complete list of *weights* provided.

The fragment analysis is a special case of weighted analysis, where only the chemical structure branch of the ontology is used. “Fragments” should be understood as molecular fragments or functional groups. Data would typically come from fragmentation mass spectrometry experiments. ChEBI roles are not relevant for the targeted use case of determining candidate structures for a measured spectrum. This analysis uses different pruning strategies on the resulting graph to highlight molecular entities that are enriched. In contrast to the standard weighted analysis option, terminal molecular leaves or root vertices are not removed.

Results are corrected for multiple hypothesis testing using Benjamini & Hochberg’s false-discovery rate, which is widely used by most enrichment analysis tools and in general whenever a hypothesis test is repeated many times. A hypothesis test implies a small risk of false positive errors occurring. For a single test the likelihood of such a false positive is normally acceptable, but the repeated execution of a test – in this case, for each class in the ontology – means that the likelihood of such an error accumulates, leading to an unacceptably high probability of false positives. That is, an ontology class may appear significantly enriched for a given input set when it is not. Multiple hypothesis testing correction mechanisms aim to reduce the risk of false positives to an acceptable level.

Currently the enrichment is calculated taking the entire ontology as background population, which is the default offered by the underlying BiNGO implementation.

The enrichment analysis may be executed with the full ChEBI ontology, although for the results visualisation BiNChE uses only the *is_a* and *has_part* relations from ChEBI’s set of relationship types. That is because the other chemical relationships included in ChEBI, such as *has_functional_parent*, are not hierarchical – they are not transitively inherited and do not directly connect molecules with chemical classes. These relationships are mostly used to capture knowledge at the leaf level, between specific molecular entities, as additional separate information to that captured in the structure-based *is_a* classification. For example, acyclovir has functional parent guanine, but while guanine is a purine nucleobase, acyclovir is not a purine nucleobase. The results display relies on the underlying transitivity of the used relationships in order to lay out the display.

The analysis can also be constrained to either the structural branch of the ChEBI ontology – which is oriented towards cheminformatics and chemical fragment based analysis use cases – or the role branch of the ChEBI ontology, catering for chemical biology enrichment analysis use cases.

Displaying data on top of ChEBI is daunting because of the size and complexity of the ontology. Using ChEBI directly with BiNGO, even after producing all the required files, results in a large and complex graph structure. To this end, we implemented pruning strategies (detailed in the wiki (https://github.com/pcm32/BiNCheWeb/wiki/BiNChE) and in Additional file 1) that, while retaining the key ontology information, remove unnecessary detail. While this can be achieved in GO-based enrichment through the use of GO Slim files, losing details of the overall knowledge organization, our tool performs pruning on-demand for each enrichment analysis, and retains the broad structure of the ontology classification. This means that chemical knowledge relevant to each particular enrichment result is retained.

**Figure 2:**
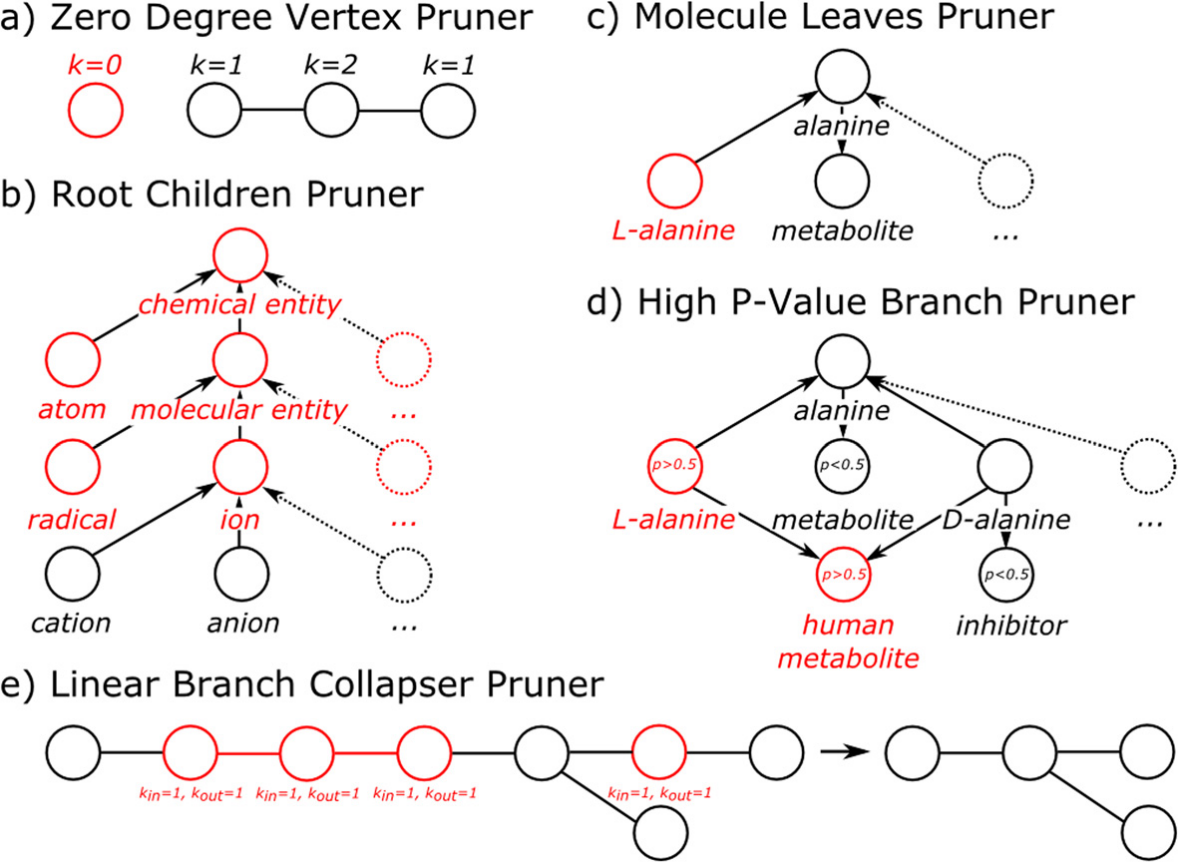
**Pruning methods used to decrease the complexity of a result graph.** Illustration of the different pruning methods implemented to reduce the complexity of the graph-based output. For all cases, edges are directed and equivalent, even if they represent different relationships. Arrows are shown where relevant. **(a)** The Zero Degree Vertex Pruner excludes nodes that have neither incoming nor outgoing edges. These normally appear once the other pruners have been applied. **(b)** The Root Children Pruner removes higher levels of the ontology that are deemed irrelevant to the enrichment analysis as they represent very general classes of objects (such as “chemical entity” or “ion”). **(c)** The Molecule Leaves Pruner removes all nodes of the graph that do not have any incoming edges and represent a distinct small molecule – and not a class of small molecules. A small molecule is defined as distinct if the node representing it has an InChI line notation. **(d)** The High p-value Branch Pruner looks for branches of the graph where only nodes with high p-value (>0.05) are present and removes those branches. **(e)** The Linear Branch Collapser Pruner finds linear stretches (nodes have only one incoming and one outgoing edge) and collapses them, leaving the nodes that surround those stretches connected.

In BiNChE, individual pruning methods are combined in different ways into pruning strategies. A pruning strategy applies the individual methods either once or iteratively. Figure 2 shows the different pruning methods. As an example, after calculating enrichment p-values, the plain enrichment analysis option available in the web application prunes the resulting ChEBI graph through the following pruning strategy: **Pre loop:** High p-value Branch Pruner (at 0.05), Linear Branch Collapser Pruner and Root Children Pruner (3 levels of depth). These are applied only once, at the beginning, in that order. **Loop:** Molecule Leaves Pruner, High p-value Branch Pruner, Linear Branch Collapser Pruner, and Zero Degree Vertex Pruner. These are applied iteratively until no change is seen in the graph. **Final run:** No pruner.

It is relevant to apply these methods iteratively because each of them changes the topology of the graph, potentially increasing the number of nodes that can be removed. While in the case of the plain enrichment analysis no pruners are used in the “Final run”, this is different for the weighted and fragment analysis. Furthermore, users of the API can change the pruning strategies by adding or removing pruning methods to each phase, to suit their needs. On a typical execution, a pruning strategy like the one described will remove somewhere between a few hundred and a few thousand nodes from the result, clearly improving the comprehensibility of the resulting visualization and allowing the researcher to focus on those parts of the ontology that are most relevant for his or her data.

## Results and discussion

The primary driving use case for developing the BiNChE tool is the need to assist and further automate the interpretation of results in metabolomics experiments. In metabolomics experiments, the small molecules in a sample of (for example) bodily fluid are identified, quantified and used to compare different experimental groups, e.g. diseased patients and healthy patients. These comparisons result in lists of small molecules that are enriched for particular groups. These molecules can then yield information about the underlying biological processes, such as metabolic pathways, that are executing differentially between the groups. Executing an enrichment analysis with the ChEBI role ontology gives information about which biological role categories are over represented in the target condition, which can yield deeper insights into mechanisms of action.

MetaboLights is a repository for cross-species, cross-technique metabolomics experiments and derived information hosted at the European Bioinformatics Institute (EBI) [10]. The study MTBLS23: “Model-driven multi-omic data analysis elucidates metabolic immunomodulators of macrophage activation” [11] contains lists of ChEBI identifiers corresponding to small molecules for which the concentration either increases or decreases during macrophage activation when presented with bacterial lipopolysaccharides (LPS). Applying plain enrichment analysis – using both structural and role ontologies as shown in the wiki (https://github.com/pcm32/BiNCheWeb/wiki/BiNChE#plain-metabolomics-of-macrophages) – to the list of ChEBI identifiers of the molecules that decrease in concentration during macrophage activation M1, reveals that there is an over representation of amino acids among the small molecules whose concentration diminishes during that activation phase, particularly in pyruvate derived amino acids, aspartate family amino acids, and serine derived amino acids.

In the study itself, the authors mention that certain amino acid biosynthesis pathways are relevant during M1 activation [11]. While the amino acid biosynthesis pathways are deemed relevant during M1 activation by the study, they are not exactly those BiNChE detects as enriched in the depletion list. The analysis provides a complementary view on the complex interplay of amino acids in the macrophage activation process.

In study MTBLS35: “Salmonella Modulates Metabolism during Growth under Conditions that Induce Expression of Virulence Genes” [12] researchers identified 66 metabolites through GC-MS that vary in concentration (decrease or increase) within Salmonella during virulence induction. Plain enrichment analysis of the complete set of small molecules shows an over representation of mostly amino-acids, fatty-acids, and nucleobases. A closer look into the data set shows that 3 sets of metabolites – within the original 66 – can be distinguised, representing: molecules with higher abundance before the induction of virulence, molecules with higher abundances 4 hours after virulence induction, and molecules with higher abundances 20 hours after virulence induction. Enrichment analysis of the first set of small molecules shows overrepresentation of nucleobases, purins, lipids and carbohydrates. The second set shows that after the first four hours of virulence, fatty acids, monosaccharides (different to those from the non-virulence phase), and amino acids have higher abundances. Finally, after 20 hours of induction, the enrichment analysis shows that small molecules in higher abundances are overrepresented in amino acids much more strongly. This use case can be inspected in detail – including lists of small molecules to submit to BiNChE – on the wiki https://github.com/pcm32/BiNCheWeb/wiki/BiNChE#plain-mtbls35-salmonella-modulates-metabolism-during-growth-under-conditions-that-induce-expression-of-virurulence-genes) and in Additional file 1.

ChEBI enrichment analysis is also useful for metabolite identification. MetFrag is an in-silico fragmentation tool for computer assisted identification of metabolite mass spectra [13]. Running MetFrag with default settings results in a list of 15 putative identifications of the fragmentation spectrum. The identifiers (available at the wiki (https://github.com/pcm32/BiNCheWeb/wiki/BiNChE#plain-metabolite-identification-through-fragments)) can be used as input for BiNChE after identifier conversion. Amongst others, plain analysis shows significant enrichment in the term *flavonoids*. This suggests that the spectrum represents a compound with a C_15_ or C_16_ skeleton. While in this example that particular information is already encoded in the molecule identifiers themselves, the advantage offered by enrichment analysis with BiNChE is that it provides the ability to highlight key information such as this in an automated fashion. In a high-throughput experiment or pipeline where hundreds or thousands of small molecules are identified, the researcher cannot be expected to examine them all manually, so these kind of insights (such as C_15_ or C_16_ skeletons being relevant within a chemical set) would only surface from an analysis like the one proposed here.

Naturally, many studies will yield a number of molecules that might not be present in ChEBI and hence not annotated to the classes of the ontology. In these cases, whatever molecules are present in ChEBI act as a sample of the complete set provided by the study or pipeline. The conclusions drawn from using BiNChE with this subset should be treated with appropriate caution considering the set submitted as a sample of the complete population of small molecules relevant to the problem. For studies that provide chemical entities through different chemical identifiers or directly via chemical structures, mapping to ChEBI identifiers can be achieved through services such as UniChem [14] or the Chemical Identifier Resolver (http://cactus.nci.nih.gov/chemical/structure). In future work, methods for automatically determining candidate class membership for novel molecules within the ChEBI ontology based on chemical structures – analogous to the automatic annotation of gene and protein identifiers to the Gene Ontology by, for example, species homology – will be developed.

The web tool can accept a list of hundreds of input ChEBI identifiers, from which only the well-defined small molecules will have an impact on the enrichment analysis. The biggest lists we have tested have been in the order of a thousand molecules, and with inputs of this size BiNChE took typically less than 10 seconds to produce a graph with which the user could interact. The interface was responsive at this level, although this will depend on the client machine used.

## Conclusions

BiNChE provides easy and highly interactive web access to enrichment analysis with the ChEBI ontology, as well as a Java standalone executable and library, which can be integrated into automated pipelines. We have given examples of typical use cases and indicated how it can help to facilitate an understanding of the biological and chemical significance of sets of metabolites, which is of particular relevance in metabolomics and systems biology.

## Availability and requirements

- **Project name:** BiNChE
- **Project home page:**http://www.ebi.ac.uk/chebi/tools/binche/
- **Operating system(s):** Platform independent
- **Programming language:** Java 1.6
- **License:** GNU GPLv3
- **Any restrictions to use by non-academics:** None.

## Additional file

Additional file 1 **Supplementary Material.**
