# Supplementary material for: BiNChE: A web tool and library for chemical enrichment analysis based on the ChEBI ontology
Source: BMC Bioinformatics. 2015 Feb 21;16(1):56. doi: 10.1186/s12859-015-0486-3 (PMC4349482; doi:10.1186/s12859-015-0486-3)
Supplement: Supplementary file 1 — Supplementary Material. [file 12859_2015_486_MOESM1_ESM.pdf]

BiNChE Supplementary Material

A more up to date and convenient format (HTML) of this material can be found online here.

BiNChE is a tool for ontology-based chemical enrichment analysis. Based on the ChEBI chemical ontology, BiNChE enables researchers to identify overrepresented, i.e. enriched, ontological terms in their data. The tool is accessible through the ChEBI website. In addition, a stand along Java library is provided here.

Following in the footsteps of enrichment tools for the Gene Ontology, BiNChE utilizes organized chemical knowledge to allow identification of chemical classes or roles or both to help analyse small molecule *omics* data. Similar to use cases in genomics, chemical enrichment analysis provides higher level information and associations, e.g. to biological roles. Enrichment analysis is an essential tool for small molecule data exploration.

Entry page: **http://www.ebi.ac.uk/chebi/tools/binche/**

- Web Interface
- Graph Pruning Strategies
- Graphical Exploration of Results
- Use Cases
- Implementation and Core Library (API)

Web Interface

Input

- Plain: The plain or unweighted analysis requires a list of ChEBI identifiers and relies on a binomial test to define whether the provided list is enriched in certain ChEBI categories.
- Weighted: For the weighted analysis, a list of ChEBI identifiers plus weights (decimal number) is needed. The ChEBI identifier and weight columns are tab-delimited. Examples for weights are intensity values from measurements or score values from putative molecule identification lists. This type of enrichment uses an implementation of the SaddleSum algorithm to calculate the significance of an enrichment.
- Fragment: This is a particular case of a weighted analysis, where only a subset of the ontology is used and certain pruners are applied. As such, the input is the same as that described for the weighted analysis.

Type of Analysis

- Plain: Plain analysis runs a binomial test to check for the statistical significance of deviations of input related ontological terms from the background population.
- Weighted: Weighted analysis runs a SaddleSum implementation that "approximates the distribution of sum of weights asymptotically by saddlepoint method" (see the manual). The weights indicate the importance of each term.
- Fragment: Fragment analysis is a weighted analysis limited to the chemical classes of the ChEBI ontology (Roles are not used) and uses different pruning strategies on the resulting graph to highlight molecular entities that are enriched. "Fragments" should be understood as molecular fragments or functional groups. Data would typically come from fragmentation mass spectrometry experiments. In contrast to the weighted analysis option, terminal molecular leaves or root vertices are *not* removed.

The significance of the results are corrected in every case for multiple hypothesis testing using Benjamini and Hochberg's false-discovery rate (FDR). In all the types of analysis, the enrichment is calculated taking the entire selected ontology as background population.

Target of Enrichment

The ChEBI chemical ontology includes three chemical branches: roles, classifications, and sub-atomic particles. BiNChE only makes use of the chemical roles and classifications. Depending on the scientific question, the branches can be used separately or in combination for an enrichment analysis.

- ChEBI structure classification: The structure classification describes a molecular entity based on its composition and/or the connectivity between its constituent atoms.
- ChEBI role classification: The role classification describes the role of a molecular entity within a biological context and/or its intended use by humans.
- ChEBI structure and role classification: The structure and role classification is the union of both classifications. Note that the structure classification is significantly larger than the role classification.

Graph Pruning Strategies

The ChEBI ontology forms a directed acyclic graph. The challenge in the visualisation of enrichment results lies in the complexity and detail of the ontology graph. An informative graph should -- first and foremost -- show enriched ontological terms. To add information to that mere list of enriched terms, it is essential to map the relative position or connectivity of those terms to each other. To avoid unnecessary cluttering of the graph, pruning strategies have been added to the graph layout to remove irrelevant terms. Only terms that are not enriched are subjected to the pruning methods. In terms of code, pruners must implement the ChEBIGraphPruner interface.

- Zero Degree Vertex Pruner: Removes vertices that have a total degree of zero.
- Root Children Pruner: Removes the first three levels of children vertices from the root vertex of the chemical and role ontology. The removed vertices refer to less meaningful terms, such as "molecular entity", "chemical substance", or "application", and skew the overall graph layout.
- Molecule Leaves Pruner: Removes leaves (terminal vertices) that represent discrete molecules and not a class or role.
- High P-Value Branch Pruner: Removes branches from the graph components that contain only vertices with a p-value greater than 0.05.
- Linear Branch Collapser Pruner: Collapses linear branches within the graph to hide connecting vertices that are not involved in branching. Consequently, these vertices have an in- and out-degree of one.

To use pruners, they need to be combined through pruning strategies. Pruning strategies implement the [PruningStrategy] (https://github.com/pcm32/BiNChE/blob/develop/src/main/java/net/sourceforge/metware/binche/graph/PrunningStrategy.java) interface. Given that the different pruners exert changes on the graph on each application, subsequent applications of them on the graph can further reduce its elements. Pruning strategies apply pruners at three stages: initial, loop, and final, which are executed in that order. For each of these stages, pruners need to be assigned (a pruner can be assigned to more than one phase). The initial and final phases only involve the application of pruners a single time, while the loop phase iterates the application of the pruners set until the graph converges. Currently, the implemented strategies are:

- Empty Pruning Strategy: No pruning applied.
- Fragment Enrichment Pruning Strategy: Applies the High P-Value Branch Pruner (with a cut-off at 0.05) and the Linear Branch Collapser Pruner, both in the initial and loop phases.
- Plain Enrichment Pruning Strategy: For the pre-loop phase this strategy applies the High Value Branch Pruner (0.05), the Linear Branch Collapser Pruner, and the Root Children Pruner (3 levels, without repetition). During the loop phase, this strategy applies the Molecule Leaves Pruner, the High P-Value Branch Pruner (0.05), the Linear Branch Collapser

Pruner, and the Zero Degree Vertex Pruner. No pruners are applied in the final phase post-loop.

- Weighted Enrichment Pruning Strategy In the initial phase, this strategy applies the Molecule Leaves Pruner, the Root Children Pruner (4 levels, no repetition), and the High P-Value Branch Pruner(0.05). No pruners are applied in the loop phase. In the final phase, the Linear Branch Collapser and Zero Degree Vertex Pruners are applied.

Graphical Exploration of Results

Once the enrichment analysis result is presented to the user through the UI, the user can explore the result interactively through the CytoscapeWeb interface provided.

- Highlighting and tool tip : hovering over a node highlights it and shows a tool tip with relevant data.

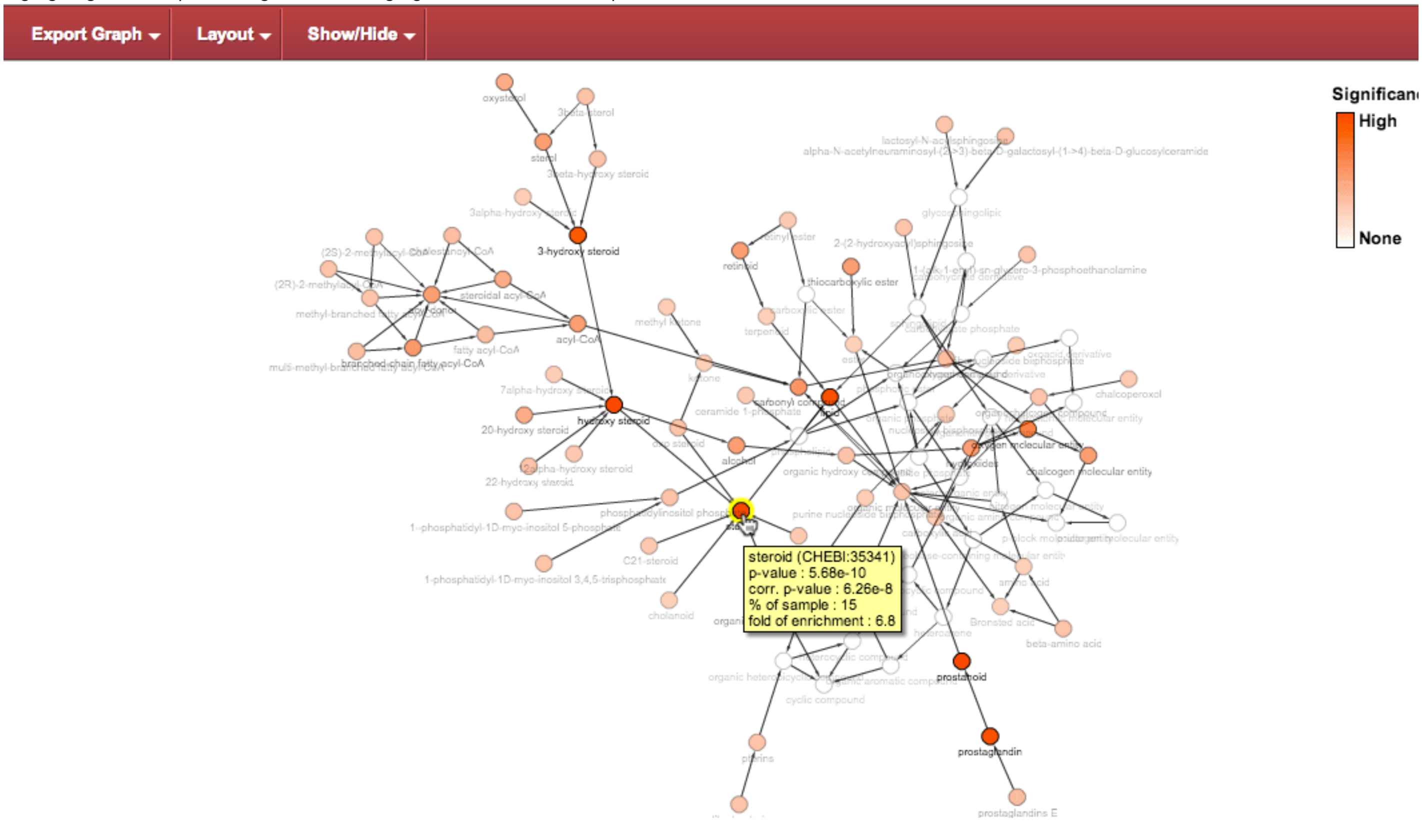

- Select node : clicking on a node selects it, changing the highlight to blue.

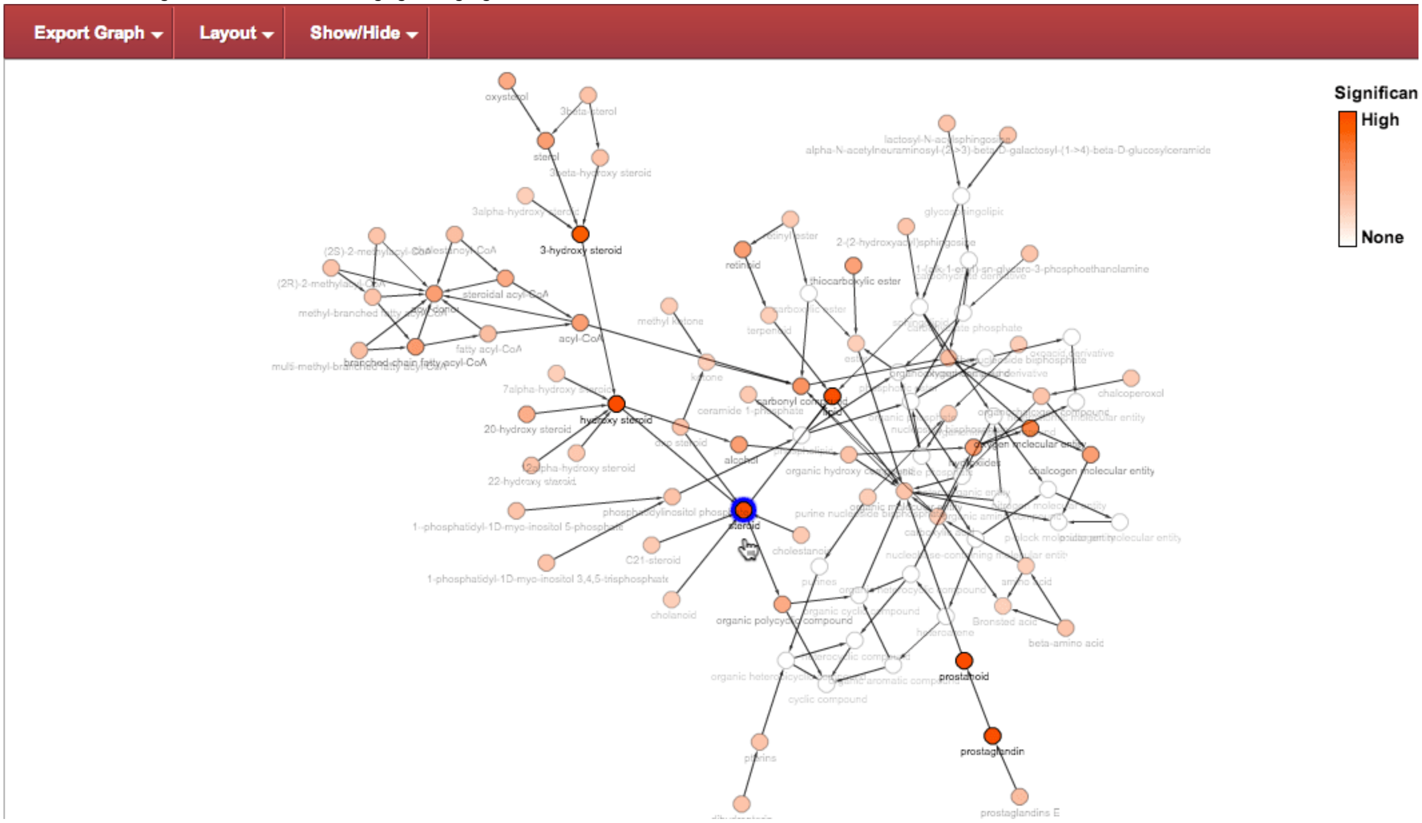

- Descendants : Given a node of interest, all its descendants can be selected through a contextual menu when clicking on the node.

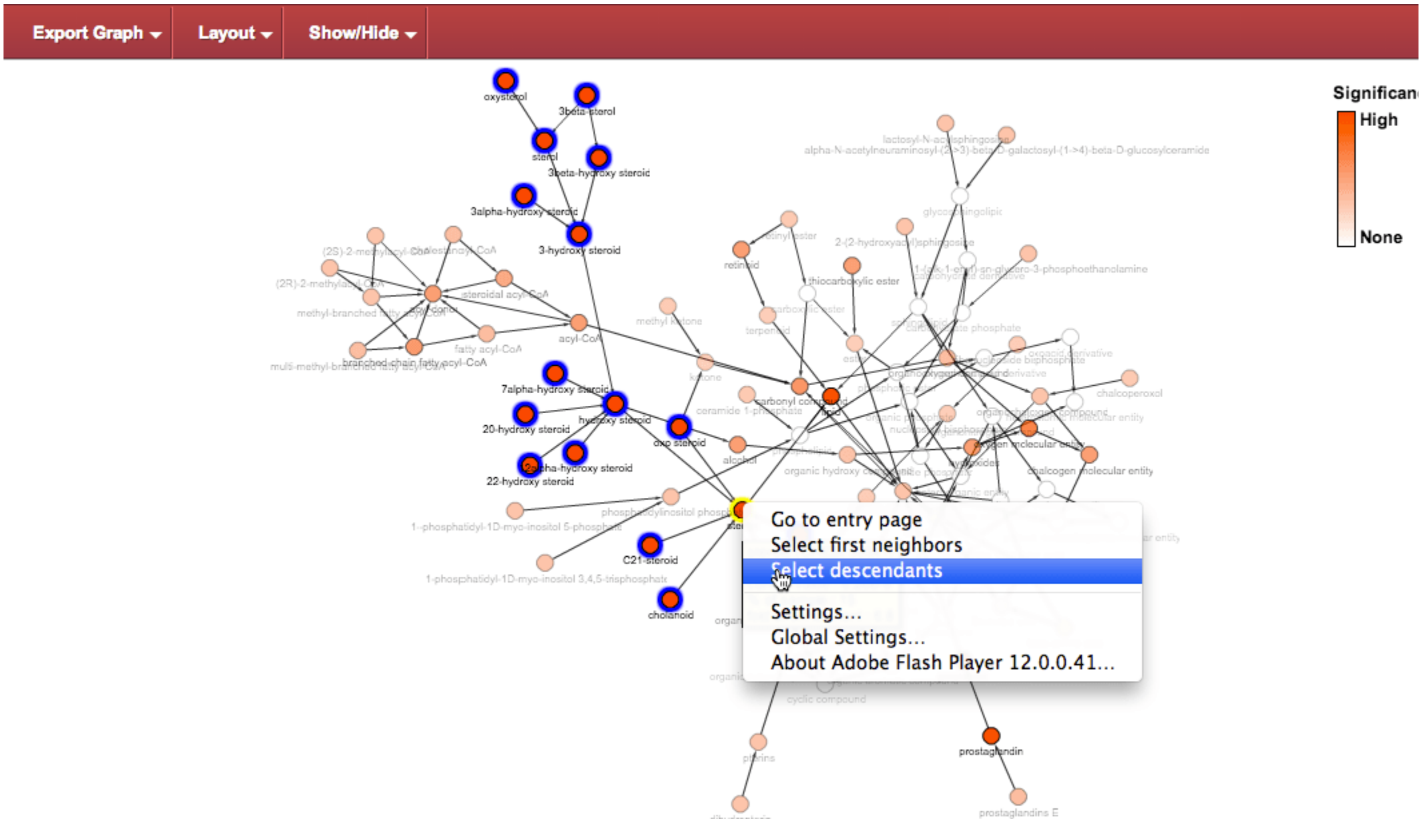

- Direct neighbours : all the connected nodes (parents and children, one degree), can be selected through a contextual menu.

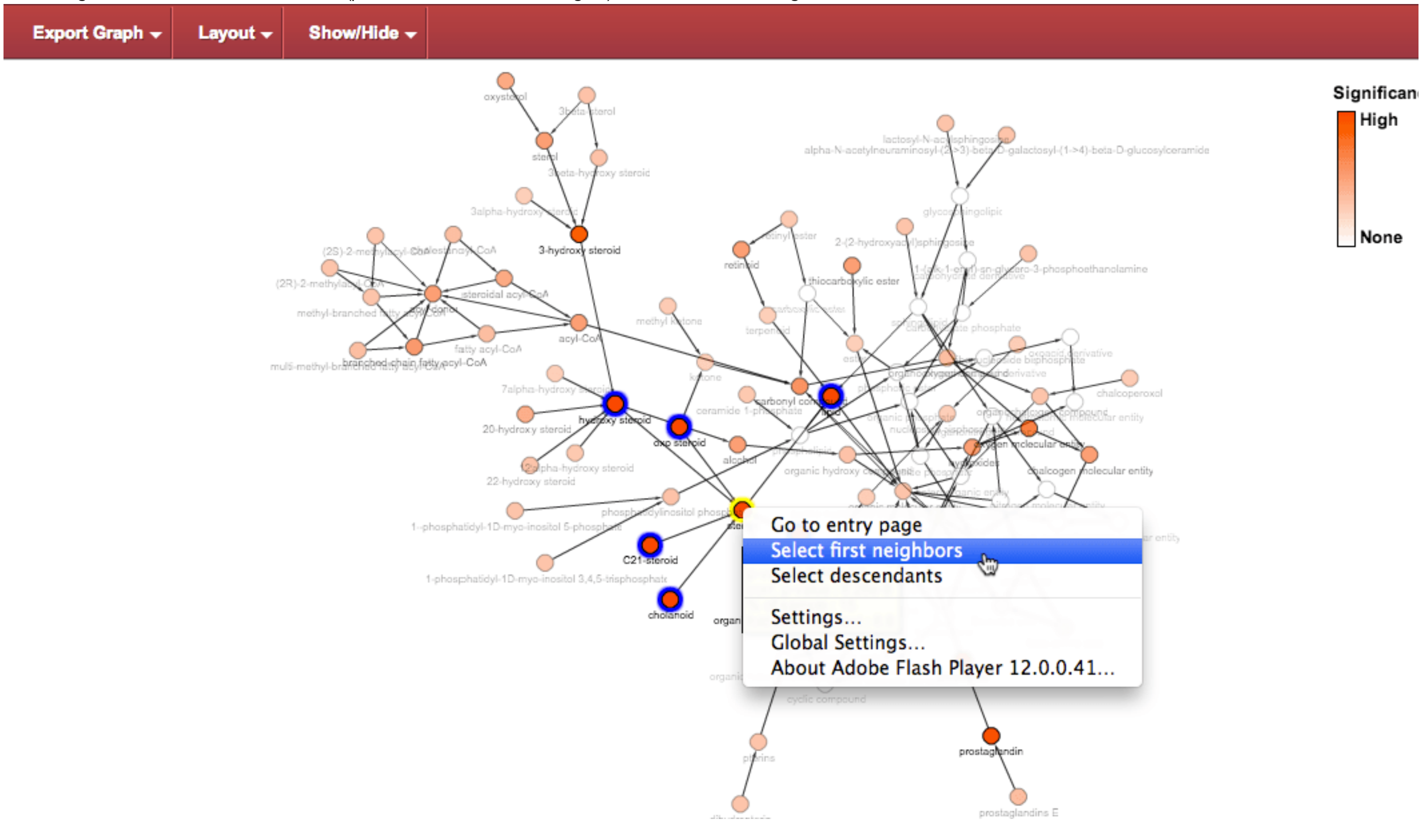

- Change layout : for the visible nodes, the layout can be changed through the menu.

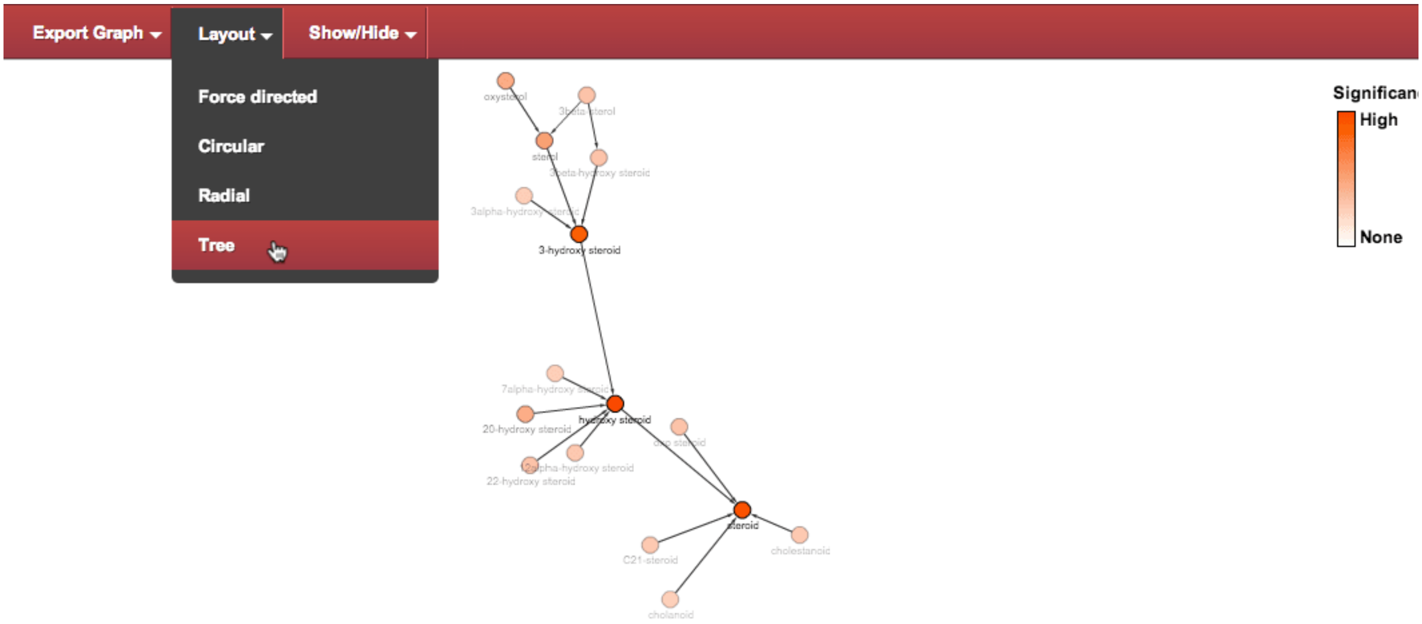

Resulting in the following arrangement for those nodes:

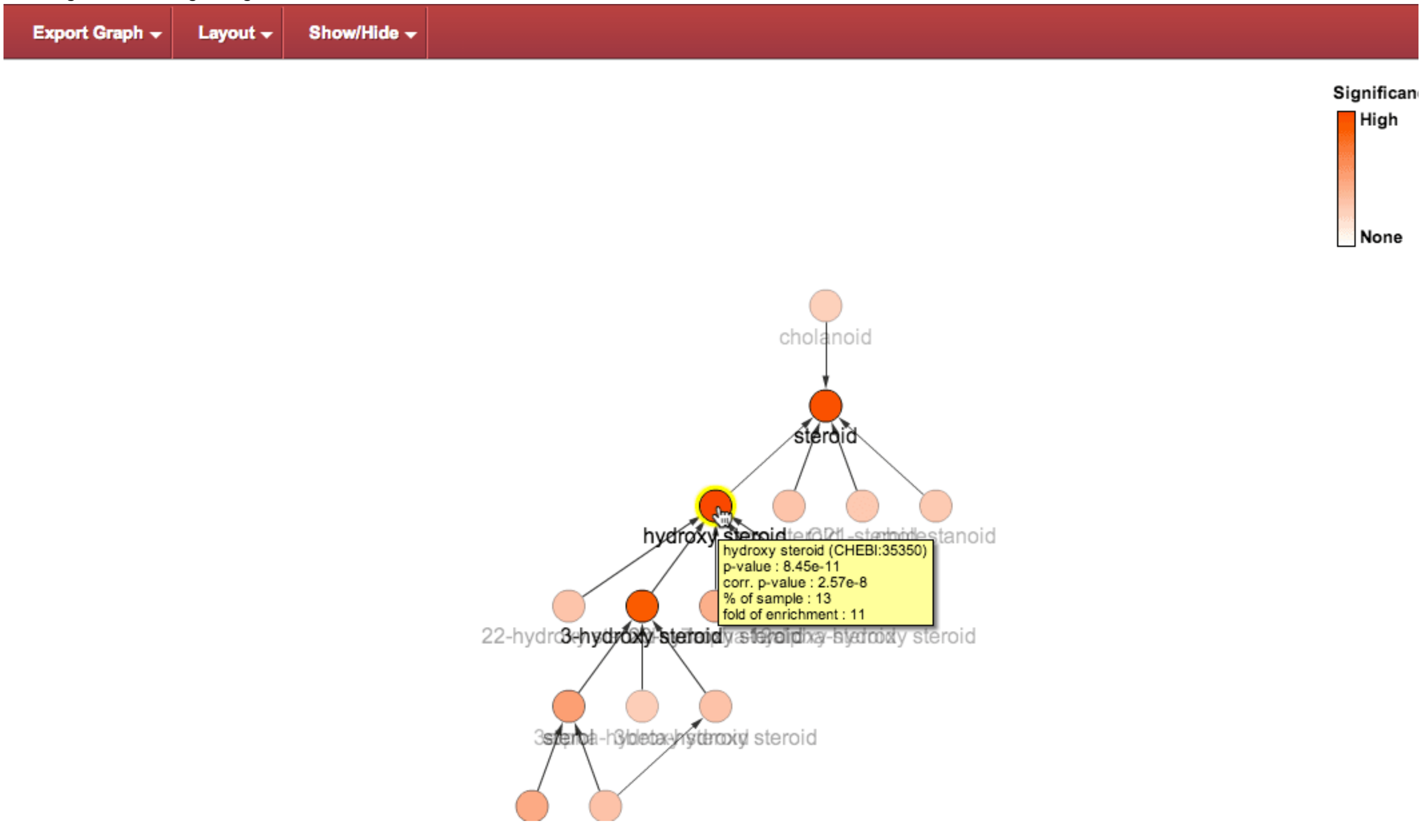

Decluttering results:

These steps allow you to declutter the graph, to focus on regions of interest.

- Hide non-selected nodes : Once a set of nodes have been selected, the complement of nodes can be hidden by using the menu:

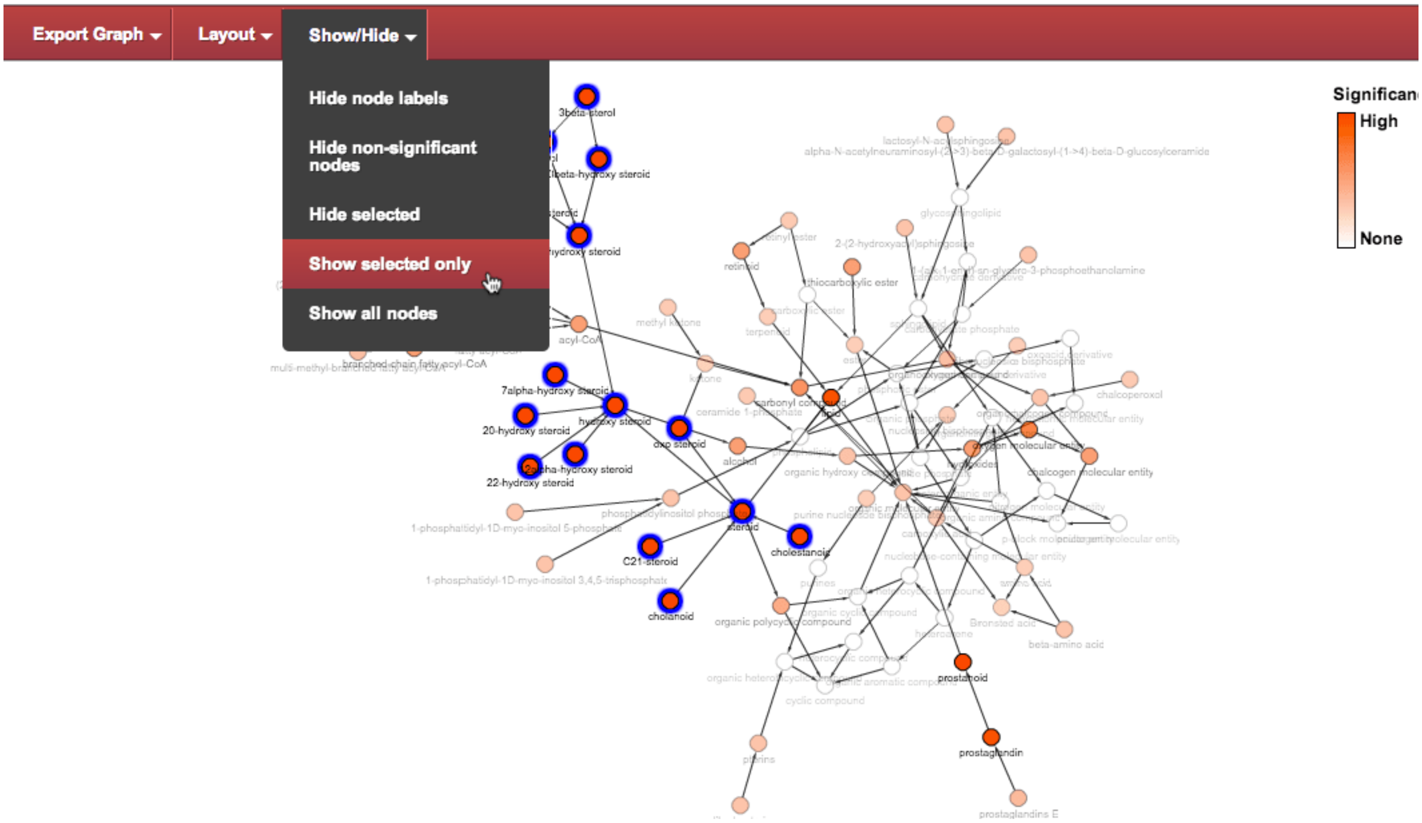

This command produces the following view:

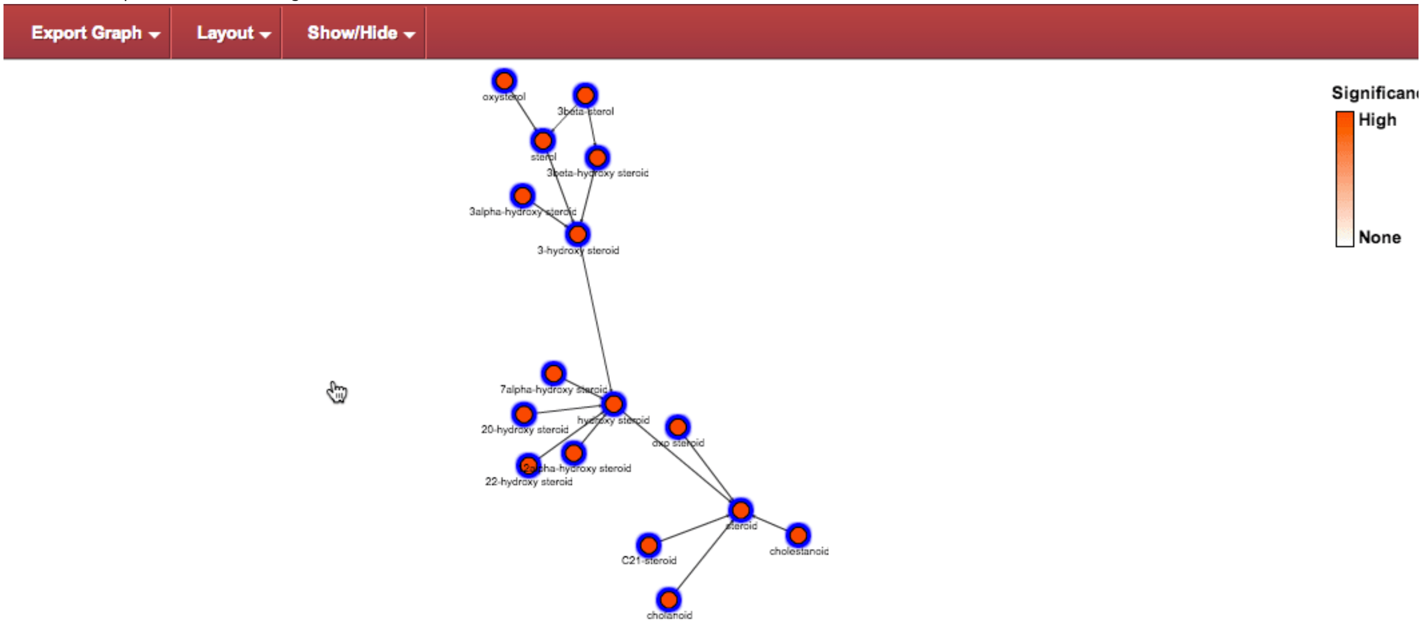

- Hide non-significant nodes : hides nodes with p-value > 0.05 through the menu:

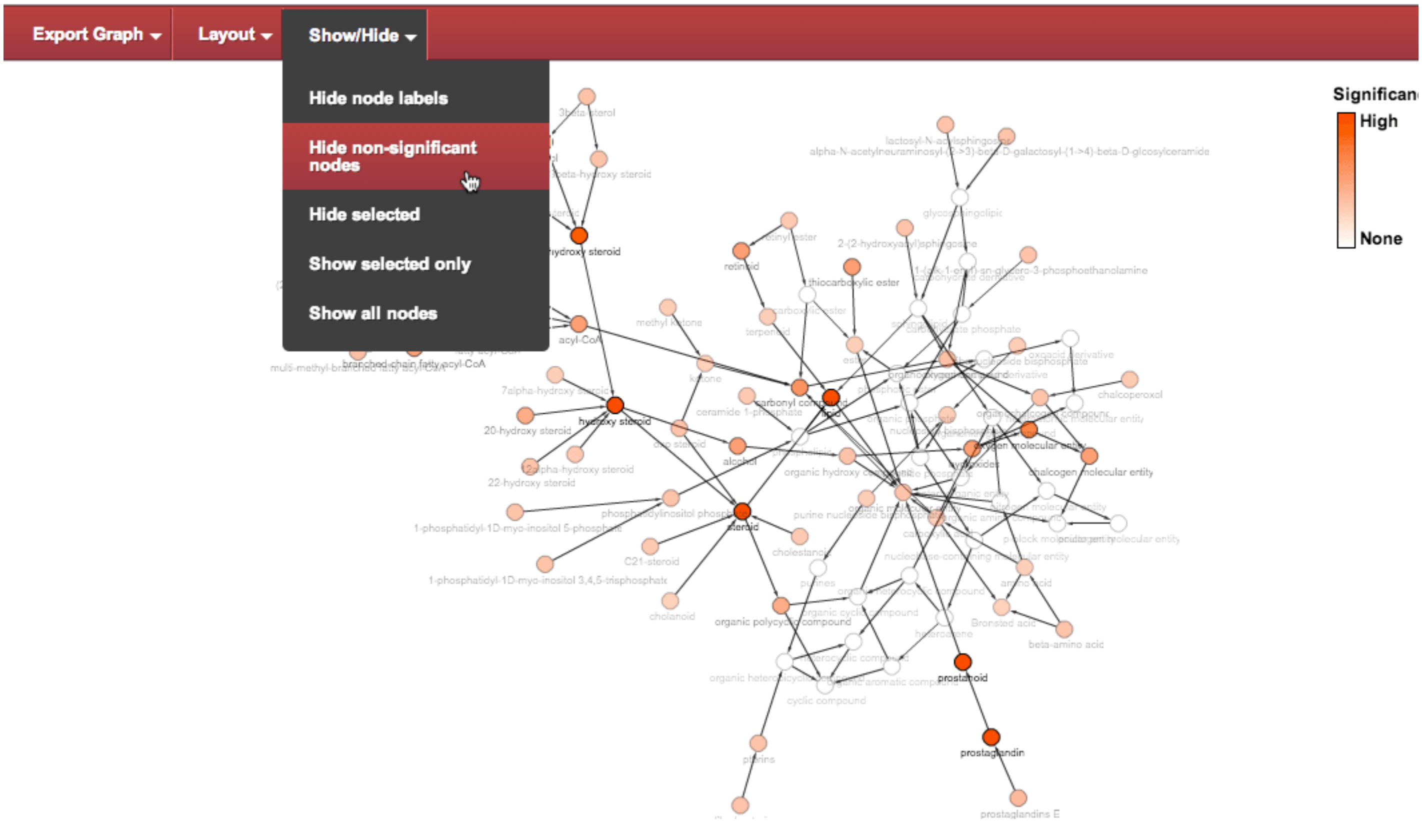

This command produces the following view:

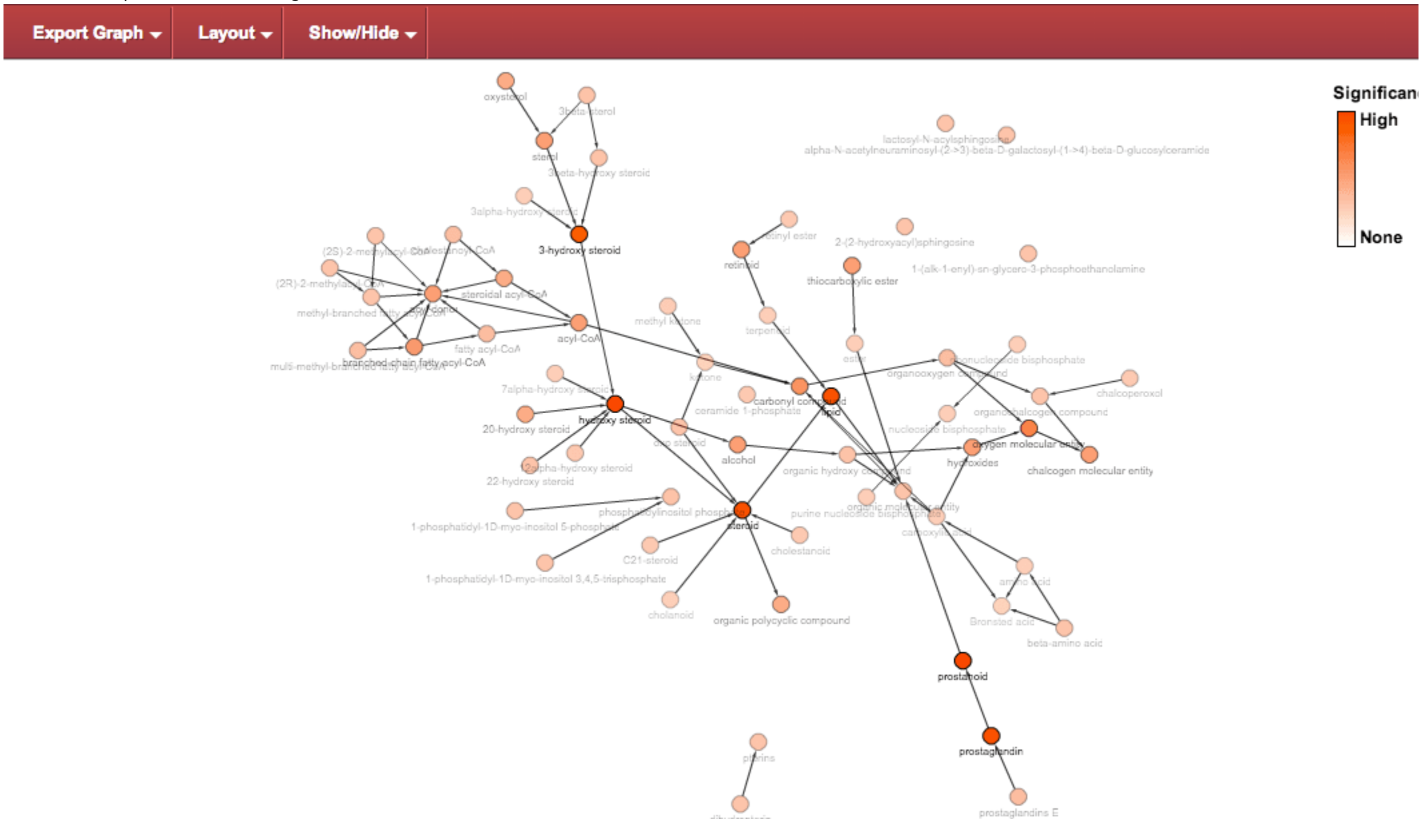

- Hide node's labels : reduces the clutter by hiding the labels of the nodes using the menu option:

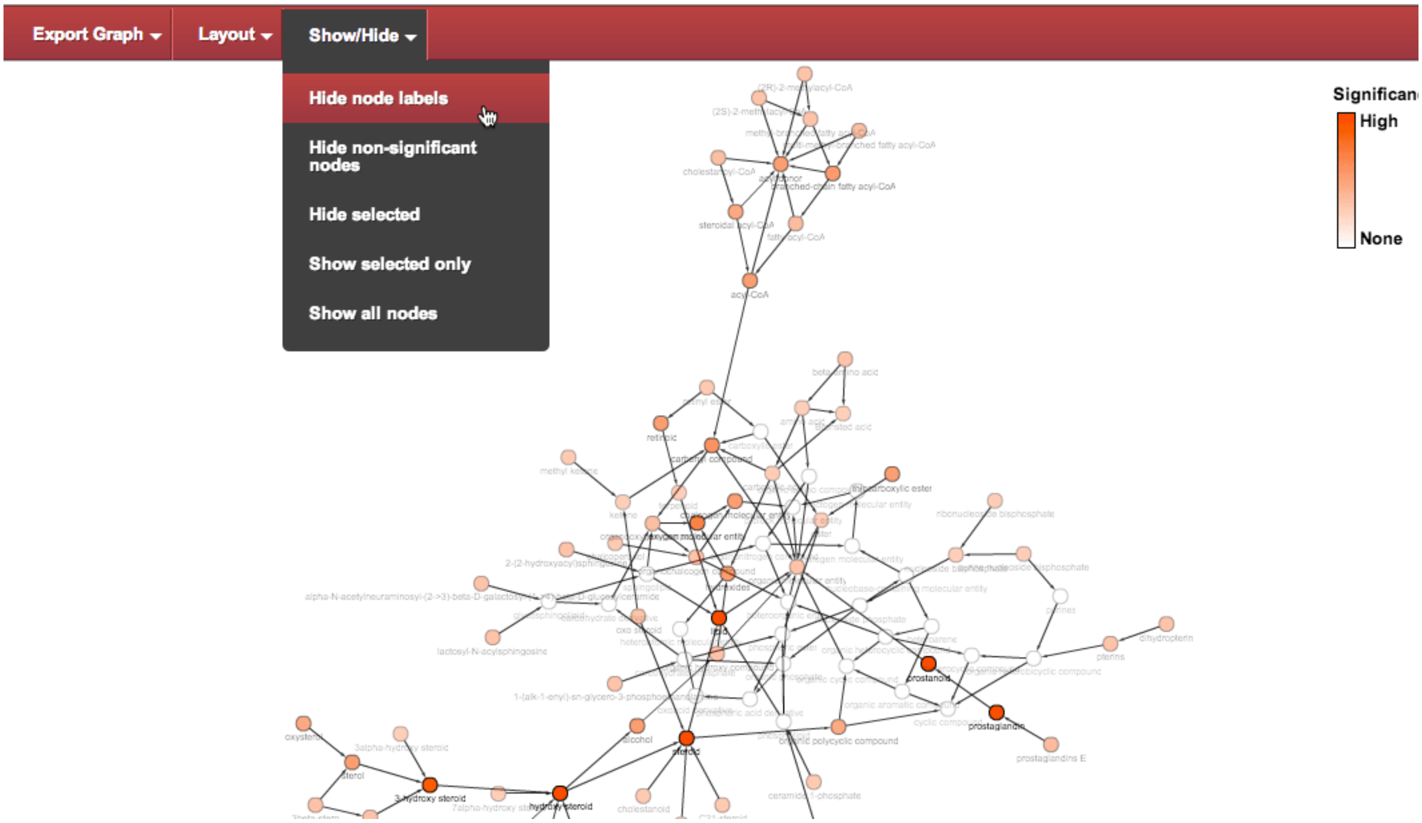

This command produces the following view:

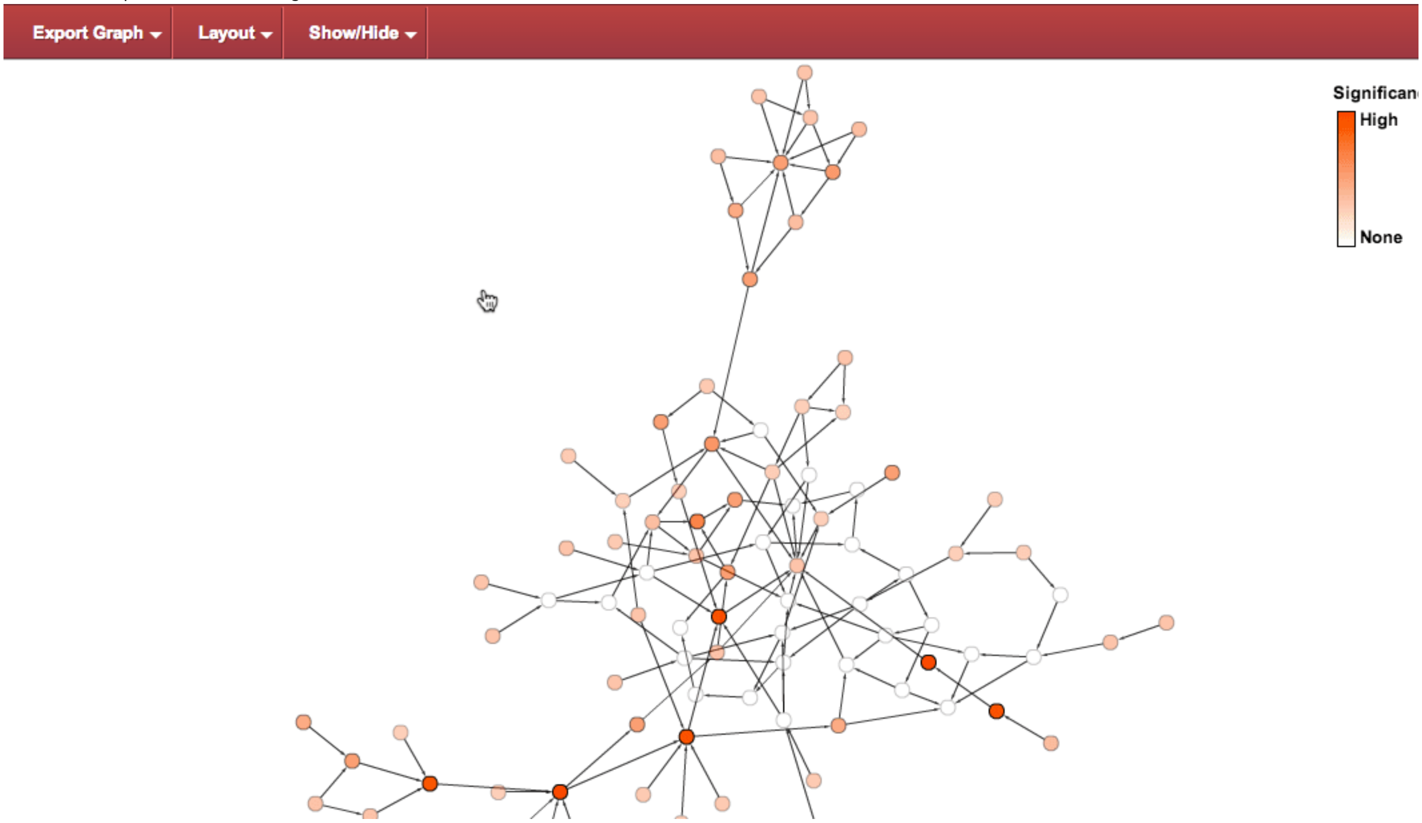

Use Cases

In general, any list of small molecules, produced via a computational pipeline, experimental technique or any other method, is suitable for the analysis through BiNChE. Examples of these could be a list of small molecules that are relevant within a set of biological assays; metabolites that are consumed or produced by a set of enzymes of interest; a set of metabolites that are known to be part of the metabolism of an organism but that are absent in other organisms of interest; a set of small molecules that where defined as relevant in a metabolomics study; etc.

Weighted

Weighted analysis provides a bird eye view of a list of compounds that have associated weights, e.g. from network analysis or metabolomics. The example below comes from an effort to build tissue specific metabolic pathways. Here, weighted enrichment analysis highlights the presence of "isoquinolinol" (CHEBI:24923) in the target tissue. Subsequent reasoning about the presence of isoquinolinols in that tissue helps to validate and refine the methods used.

```
CHEBI:17079    0.7665
CHEBI:46816    0.7465
CHEBI:28658    0.7465
CHEBI:28611    0.7465
CHEBI:28594    0.6915
CHEBI:17048    0.6915
CHEBI:7852    0.60575
CHEBI:164200    0.2342
CHEBI:8489    0.25321
CHEBI:9630    0.2543
CHEBI:59477    0.2335
CHEBI:9495    0.2433
CHEBI:3540    0.509
```

Plain - Metabolite identification through fragments

Plain analysis can be used to analyse metabolite identification lists from MetFrag. Running MetFrag with default settings results in a list of 15 putative identifications of the fragmentation spectrum. The identifiers can be used as input for BiNChE after identifier conversion (e.g. using the ChEBI plug-in in KNIME). Amongst others, plain analysis shows significant enrichment in the term *flavonoids*. This suggests that the spectrum represents a compound with a C15 or C16 skeleton.

```
CHEBI:78023
CHEBI:78026
CHEBI:78029
CHEBI:15649
CHEBI:34707
CHEBI:8908
CHEBI:52047
CHEBI:27587
CHEBI:28103
CHEBI:17846
CHEBI:27725
CHEBI:18131
CHEBI:16035
CHEBI:3237
CHEBI:15413
```

Plain - Metabolomics of macrophages

Metabolights is a repository of metabolomics experiments. The study MTBLS23: "Model-driven multi-omic data analysis elucidates metabolic immunomodulators of macrophage activation" contains a list of small molecules with ChEBI identifiers that increase or decrease during the macrophage activation process. If we use the list of ChEBI entities that decrease with both the *role and structural ontologies*, we can see that, as the figure below shows, aminoacids are most relevant in this part of the study.

```
CHEBI:16797
CHEBI:17141
CHEBI:15728
CHEBI:18344
CHEBI:16335
CHEBI:16027
CHEBI:16958
CHEBI:30769
CHEBI:16737
CHEBI:27389
CHEBI:10696
CHEBI:30796
CHEBI:52742
CHEBI:48300
CHEBI:15428
CHEBI:17345
CHEBI:16668
CHEBI:17368
CHEBI:17596
CHEBI:13172
CHEBI:16977
CHEBI:17053
CHEBI:16015
CHEBI:15603
CHEBI:18019
CHEBI:15729
CHEBI:17115
CHEBI:16857
CHEBI:16414
CHEBI:16995
CHEBI:15756
CHEBI:46905
CHEBI:8337
CHEBI:32816
CHEBI:16610
CHEBI:15746
CHEBI:28842
CHEBI:16695
CHEBI:17712
```

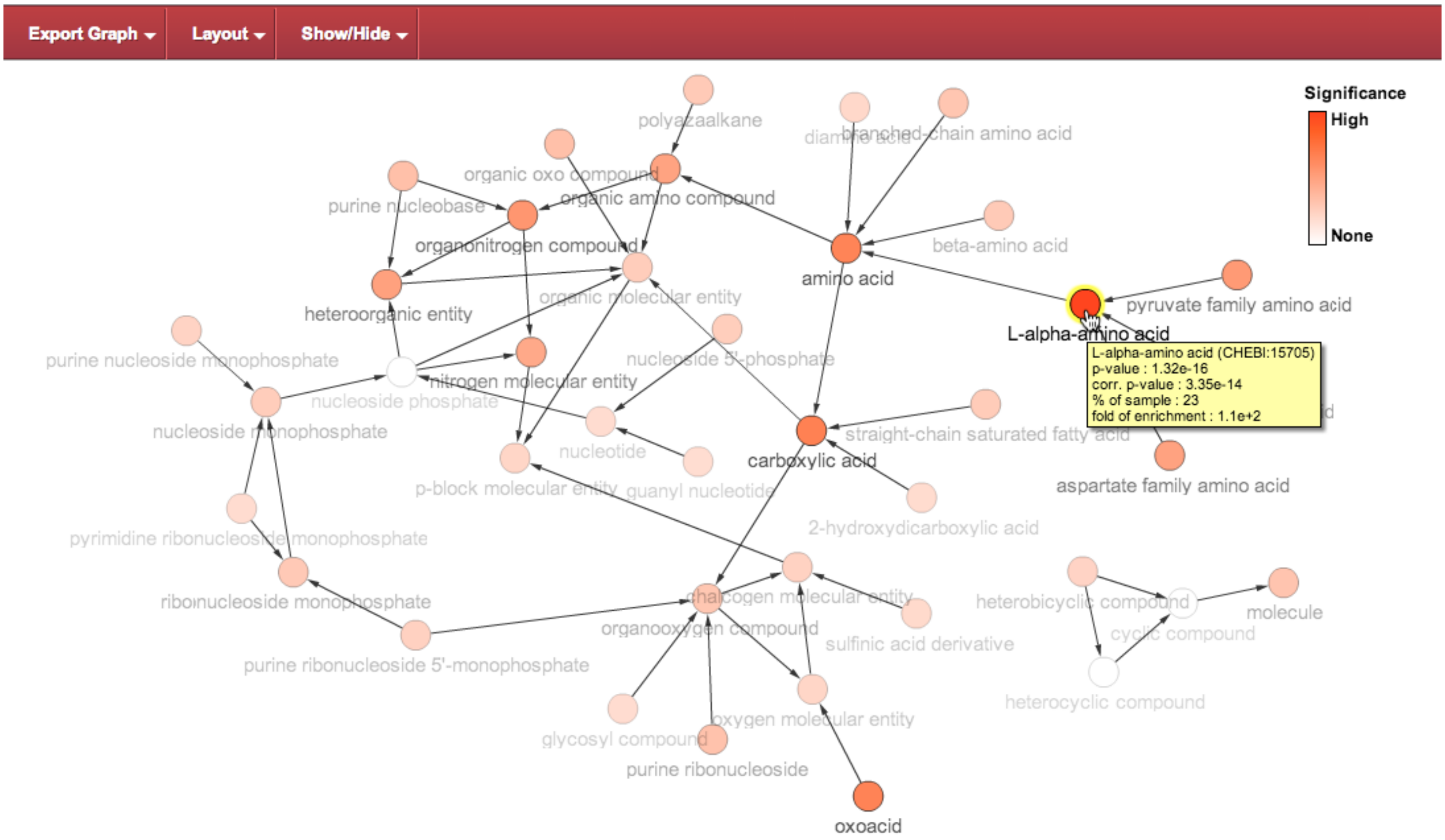

Plain - MTBLS35: Salmonella Modulates Metabolism during Growth under Conditions that Induce Expression of Virulence Genes

The MetaboLights study MTBLS35 shows the metabolic response of Salmonella when virulence genes are induced. In this study, the researchers identified 66 metabolites that change (either decrease or increase) during virulence induction through GC-MS (see original work here). The list of metabolites, obtained from the MetaboLights site, and mapped to ChEBI using the Batch Conversion tool from the FiehnLab is available here.

The list of 66 metabolites (as ChEBI IDs) is:

```
CHEBI:26078
CHEBI:44897
CHEBI:29888
CHEBI:17148
CHEBI:32816
CHEBI:422
CHEBI:28875
CHEBI:16610
CHEBI:15741
CHEBI:17821
CHEBI:17568
CHEBI:16199
CHEBI:27248
CHEBI:17712
CHEBI:15699
CHEBI:16335
CHEBI:15918
CHEBI:16708
CHEBI:16958
CHEBI:16015
CHEBI:15725
CHEBI:32398
CHEBI:28140
CHEBI:29003
CHEBI:15954
CHEBI:59265
CHEBI:16196
CHEBI:15728
CHEBI:28842
CHEBI:15760
CHEBI:17561
CHEBI:16977
CHEBI:17115
CHEBI:17053
CHEBI:18019
CHEBI:16040
CHEBI:28645
CHEBI:17895
CHEBI:15603
CHEBI:16643
```

```
CHEBI:17203
CHEBI:4167
CHEBI:15729
CHEBI:15971
CHEBI:27266
CHEBI:16857
CHEBI:46905
CHEBI:16108
CHEBI:8337
CHEBI:16027
CHEBI:17533
CHEBI:15428
CHEBI:15693
CHEBI:15850
CHEBI:15978
CHEBI:17375
CHEBI:17368
CHEBI:24898
CHEBI:23673
CHEBI:30794
CHEBI:30797
CHEBI:17385
CHEBI:15940
CHEBI:16349
CHEBI:15756
CHEBI:47013
```

Submitting this list to BiNChE for structural and role analysis, we obtain the following output:

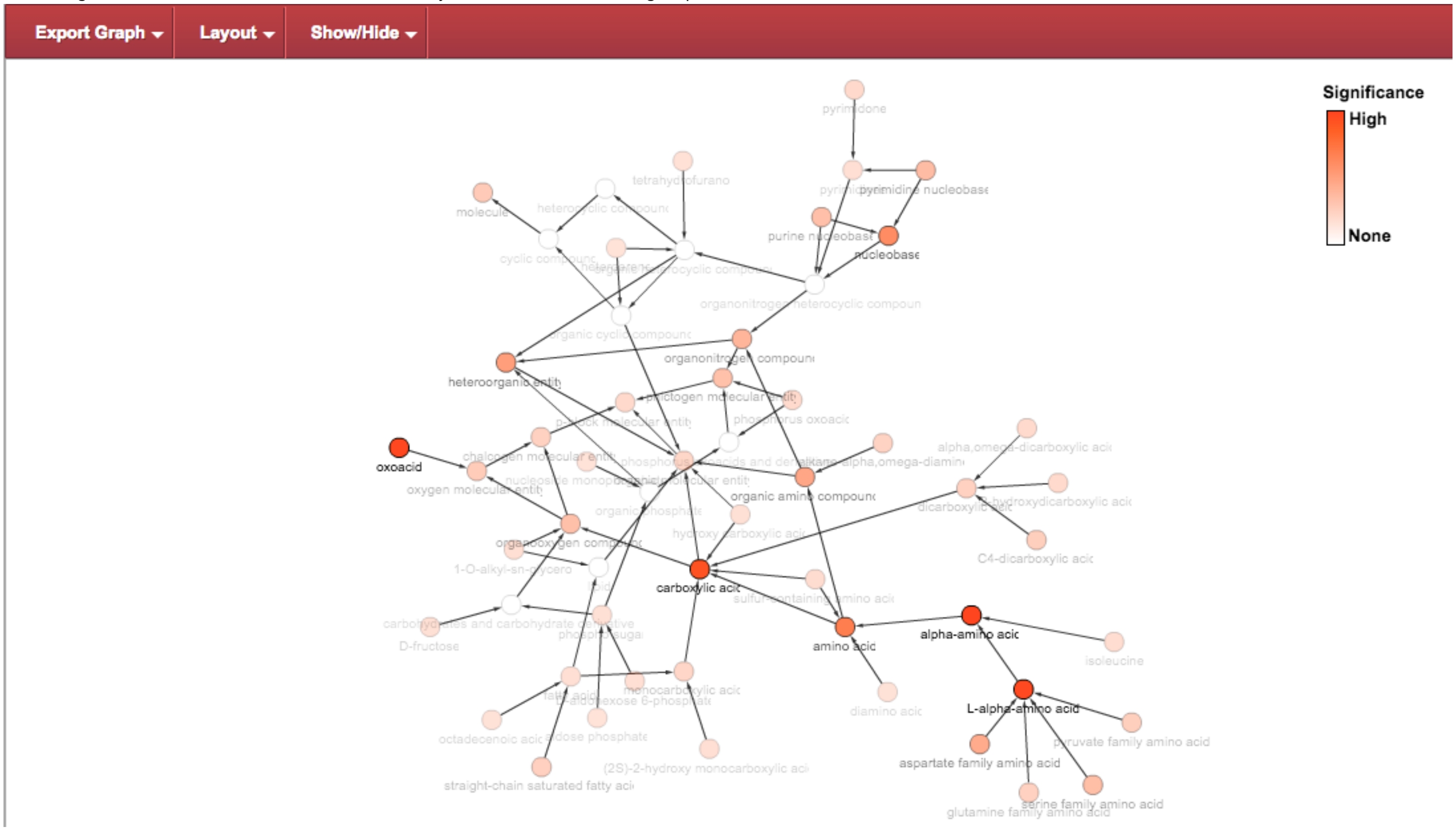

which shows an enrichment of amino-acids, fatty-acids, and nucleobases. However, this first result includes metabolites that are relevant for both the control condition and the virulence state, we would like to separate this set of metabolites according to what is relevant in each state. In the study, they detected metabolites through GC-MS in non-virulent state (Salmonella cultured on LB media, control), 4 hours after virulence induction (Salmonella move to LPM media, sample taken 4 hours later), and 20 hours after virulence induction (same LPM media). They produced four replicates for each state. Using the following R code

```
library(data.table)
fread("dataForSalmonellaUseCase.txt")->salmonella
as.matrix(salmonella[,c(7:24),with=F])->salmonella.mat
rownames(salmonella.mat)<-salmonella$ChEBI
colnames(salmonella.mat)<-colnames(salmonella[,c(7:24),with=F])

library(gplots)

dist(salmonella.mat,method = "euclidean")->salmonella.mat.dist
hclust(salmonella.mat.dist,method = "centroid")->salmunella.mat.hclust
breaks = seq(min(salmonella.mat,na.rm = T),max(salmonella.mat,na.rm = T),length.out=1000)
gradient1 = colorpanel( sum( breaks[-1]<=0 ), "blue", "white" )
gradient2 = colorpanel( sum( breaks[-1]>0 ), "white", "red" )
hm.colors = c(gradient1,gradient2)
heatmap.2(salmonella.mat,dendrogram = "row",Colv = FALSE, breaks = breaks, col=hm.colors, labRow = c(""))->salmonella.hm2
```

```
color_clusters(salmonella.hm2$rowDendrogram,k=3,groupLabels = T)->salmonella.hm2.colours
heatmap.2(salmonella.mat, dendrogram = "row", Colv = FALSE, breaks = breaks, col=hm.colors,Rowv = salmonella.hm2.colours, labRow = c(""))-
>salmonella.hm2.coloured

#plot(salmonella.hm2.colours)
slice(salmonella.hm2.colours,k = 3)->chebiGroups
write.table(file="group1ChEBISalmonellaUseCase.txt",names(chebiGroups[chebiGroups==1]),quote = F,row.names = F, col.names = F)
write.table(file="group2ChEBISalmonellaUseCase.txt",names(chebiGroups[chebiGroups==2]),quote = F,row.names = F, col.names = F)
write.table(file="group3ChEBISalmonellaUseCase.txt",names(chebiGroups[chebiGroups==3]),quote = F,row.names = F, col.names = F)
```

plus the [data file](#) from the supplementary materials available at MetaboLights (included with this wiki), one can replicate Figure 2 of the paper:

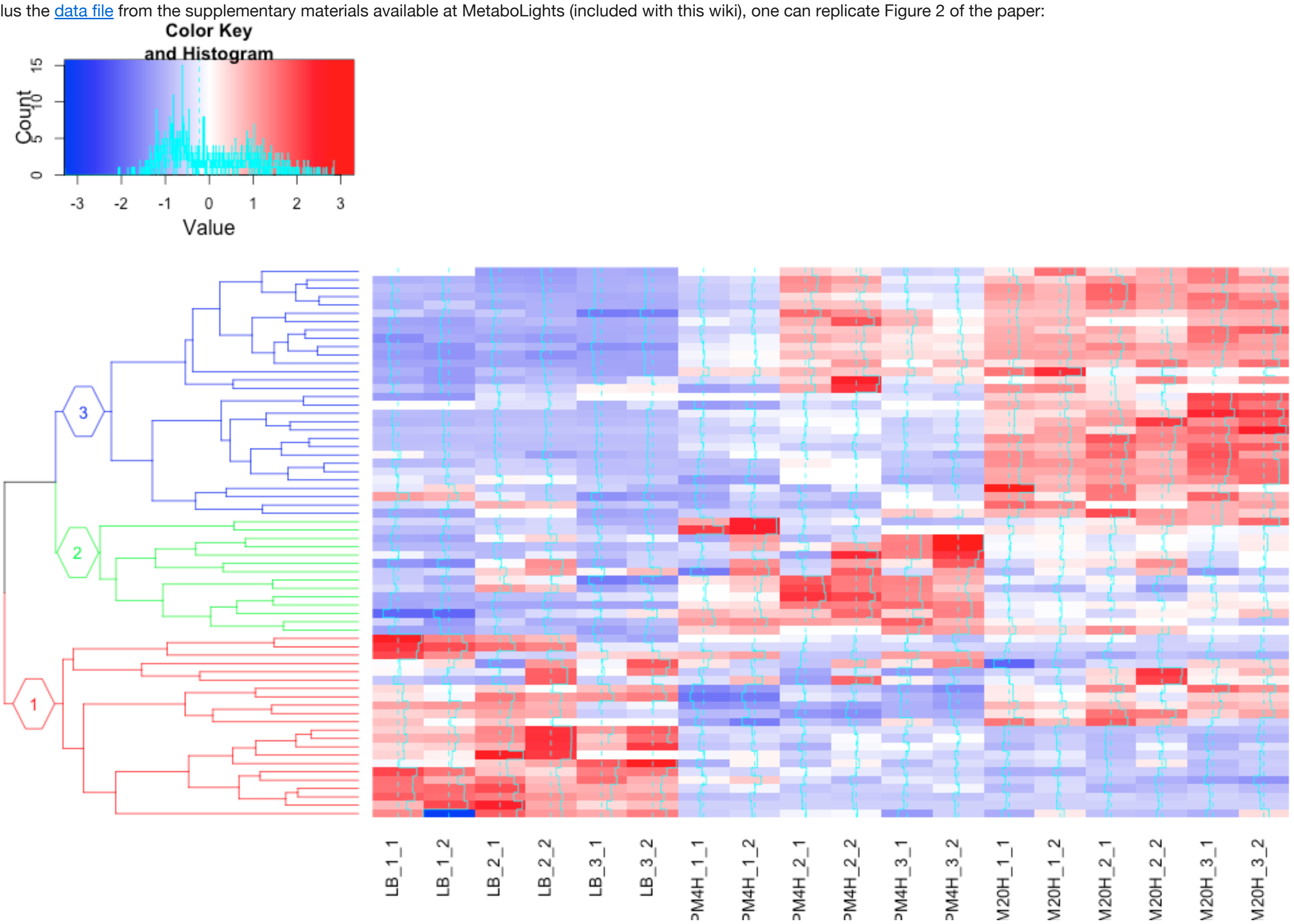

From the heatmap, 3 groups of ChEBI entities can be separated (rows of the heatmap; columns are the conditions), as the colours of the dendrogram to the left of the heatmap indicates:

1.- Metabolites with higher abundance in control (non-virulence) and lower abundance during virulence induction (both at 4 hours and at 20 hours after induction):

```
CHEBI:16958
CHEBI:16108
CHEBI:16610
CHEBI:30794
CHEBI:32816
CHEBI:59265
CHEBI:16196
CHEBI:28645
CHEBI:17375
CHEBI:15954
CHEBI:15725
CHEBI:28875
CHEBI:16027
CHEBI:16708
CHEBI:15940
CHEBI:15760
CHEBI:8337
CHEBI:16349
CHEBI:26078
CHEBI:15693
CHEBI:15978
CHEBI:15850
```

2.- Metabolites with higher abundance during first 4 hours after induction of virulence (and lower abundance in control and 20 hours after induction of virulence)

```
CHEBI:16199
CHEBI:16857
CHEBI:15741
```

```
CHEBI:27266
CHEBI:30797
CHEBI:17368
CHEBI:17568
CHEBI:15756
CHEBI:28842
CHEBI:422
CHEBI:4167
CHEBI:29003
CHEBI:15699
CHEBI:47013
```

3.- Metabolites with higher abundance after 20 hours of virulence induction (but lower in control and 4 hours after virulence induction)

```
CHEBI:17533
CHEBI:16335
CHEBI:44897
CHEBI:29888
CHEBI:15918
CHEBI:16040
CHEBI:16015
CHEBI:17712
CHEBI:17561
CHEBI:18019
CHEBI:15971
CHEBI:17115
CHEBI:32398
CHEBI:23673
CHEBI:28140
CHEBI:15728
CHEBI:24898
CHEBI:46905
CHEBI:27248
CHEBI:17148
CHEBI:15428
CHEBI:16977
CHEBI:17053
CHEBI:17385
CHEBI:17821
CHEBI:15603
CHEBI:17895
CHEBI:15729
CHEBI:16643
CHEBI:17203
```

Submitting set 1 shows the following enrichment:

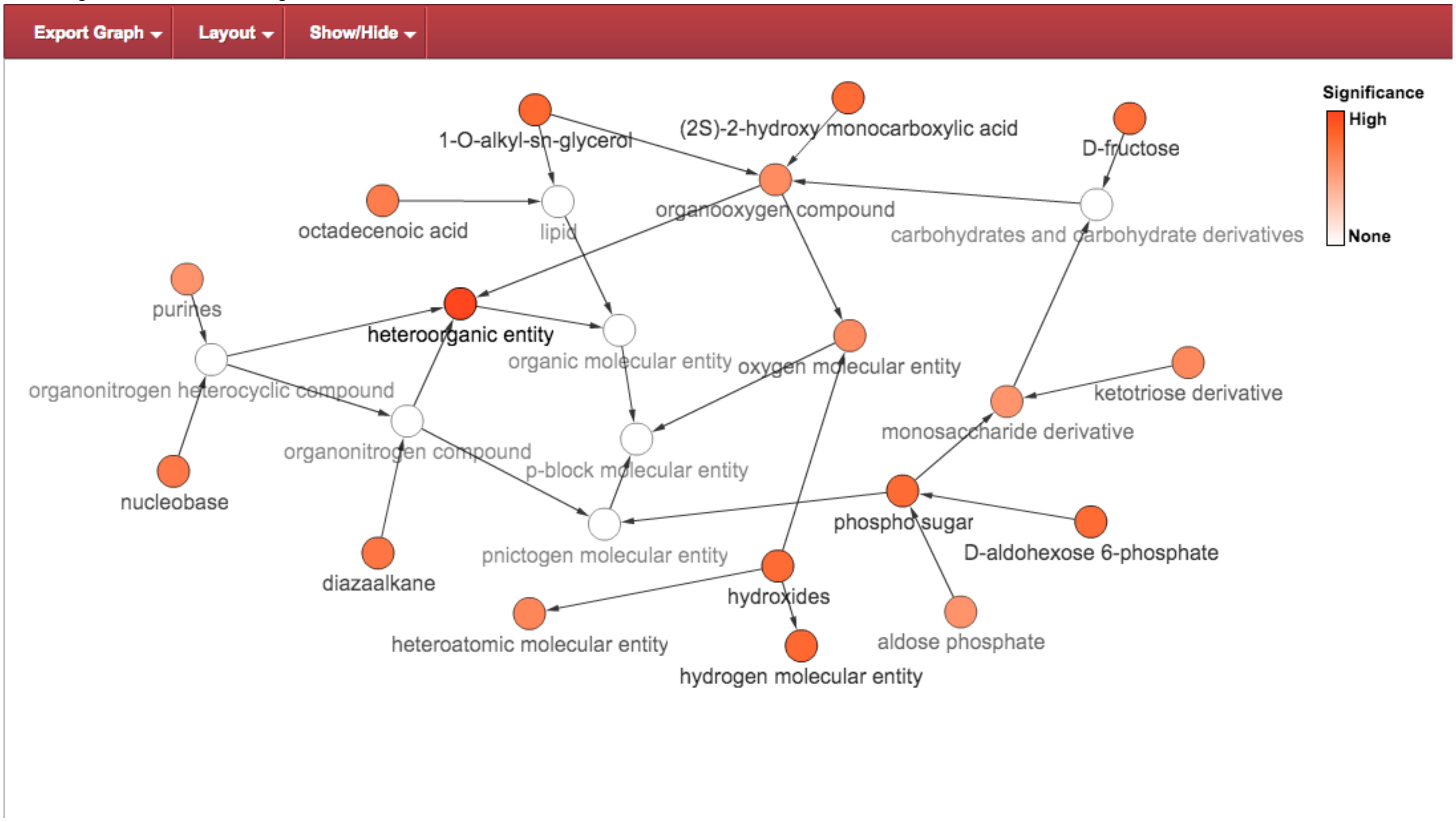

Which shows that in the non-virulent phase, nucleobases, purins, lipids and carbohydrates are relevant.

Submitting set 2 shows the following enrichment:

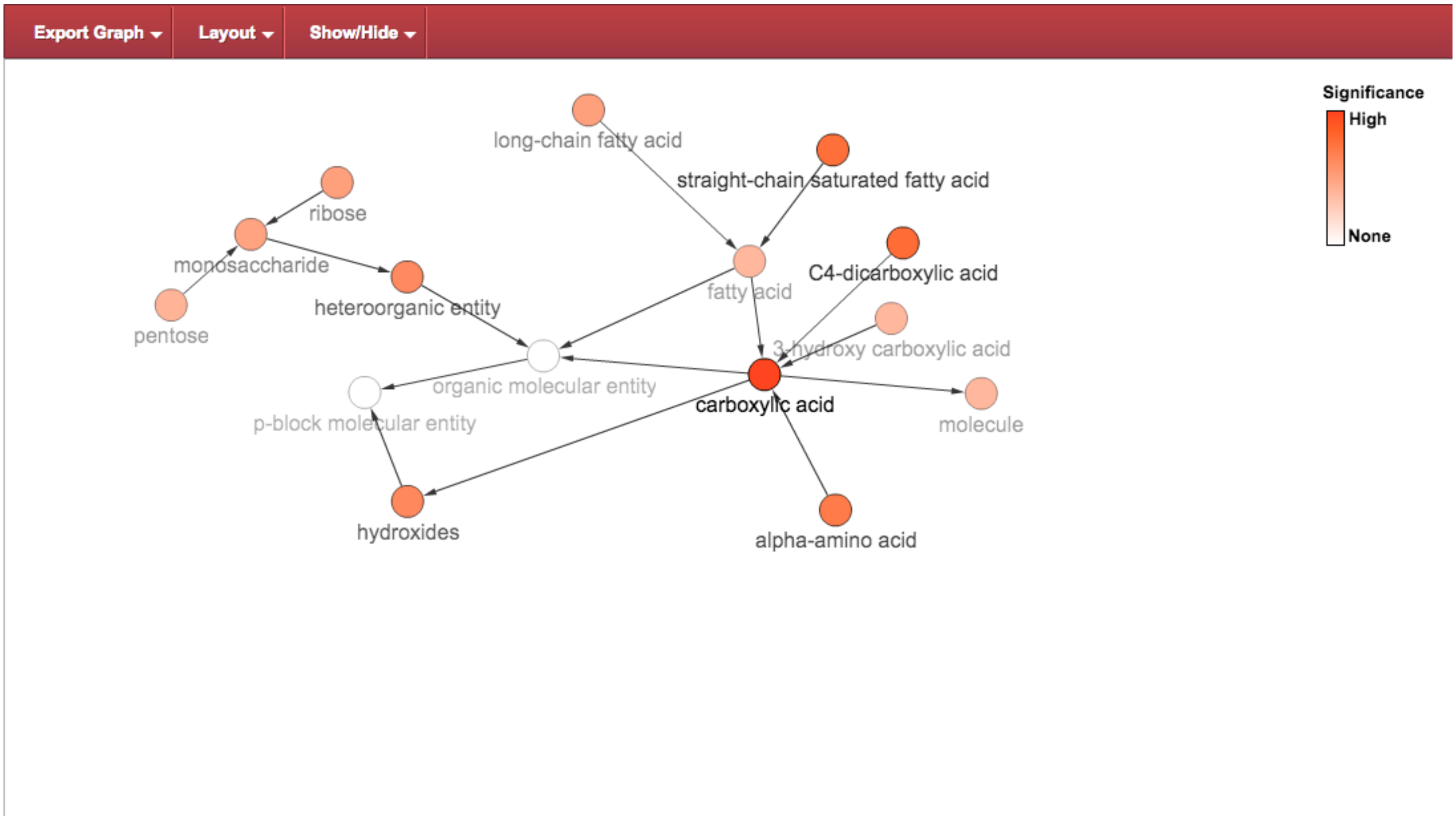

This implies that during the first four hours of virulence, fatty acids, some different monosaccharides (to the ones on the non-virulence phase), and alpha-amino acids start to play a role.

Submitting set 3 shows the following enrichment:

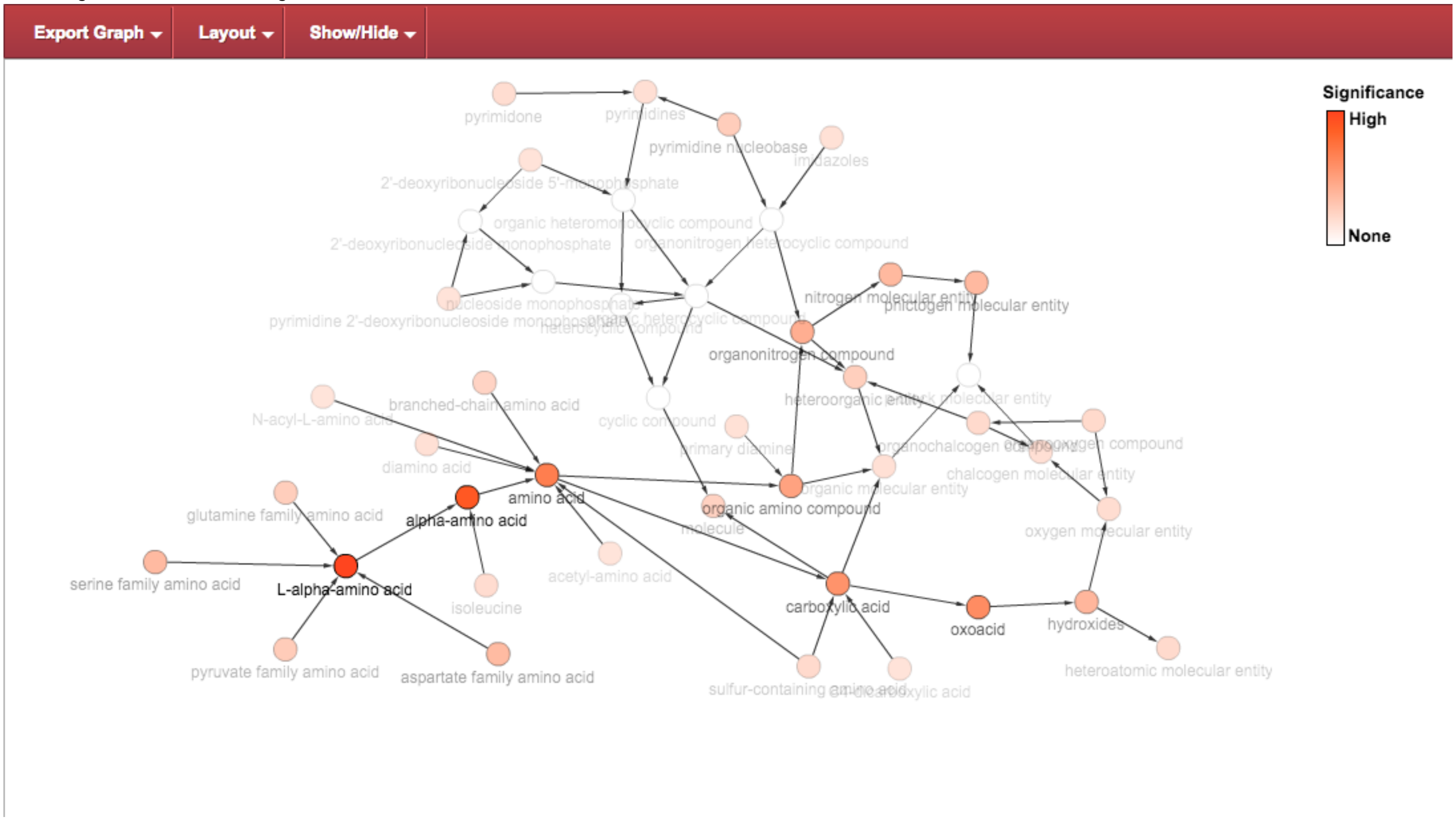

Portraying that at 20 hours after the induction of virulence, higher abundances are shifted towards amino acids.

Implementation and Core Library (API)

Source code for the core library can be found here. Javadocs for the core API can be found here. An example of usage would be:

```
Preferences binchePrefs = Preferences.userNodeForPackage(BiNChe.class);
try {
    if (binchePrefs.keys().length == 0) {
        // loads the ChEBI Ontology file from ChEBI and process it for BiNChE
```

```java
        // if this hasn't been done already.
        new OfficialChEBIOboLoader();
    }
} catch (Exception e) {
    LOGGER.error("Problems loading preferences", e);
    return;
}

String ontologyFile = binchePrefs.get(BiNChEOntologyPrefs.RoleAndStructOntology.name(), null);
// the input path points to a file where the list of ChEBI IDs (one per line, CHEBI:03432) are stored.
String elementsForEnrichFile = inputPath;

LOGGER.log(Level.INFO, "Setting default parameters ...");
BingoParameters bingoParameters = getDefaultParameters(ontologyFile);

BiNChe binche = new BiNChe();
binche.setParameters(bingoParameters);

LOGGER.log(Level.INFO, "Reading input file ...");
try {
    binche.loadDesiredElementsForEnrichmentFromFile(elementsForEnrichFile);
} catch (IOException e) {
    LOGGER.log(ERROR, "Error reading file: " + e.getMessage());
    System.exit(1);
}

// enrichment analysis execution.
binche.execute();

// object to receive and process results
ChebiGraph chebiGraph =
        new ChebiGraph(binche.getEnrichedNodes(), binche.getOntology(), binche.getInputNodes());
// the ChebiGraph can be traversed, for instance, to make a table of enrichment.

LOGGER.log(Level.INFO, "Writing out graph ...");
SvgWriter writer = new SvgWriter();
// the graph can be written to svg.
writer.writeSvg(chebiGraph.getVisualisationServer(), outputPath);
```

http://cytoscapeweb.cytoscape.org/ https://github.com/pcm32/BiNChE
